# FUS-ERG induces late-onset azacitidine resistance in acute myeloid leukaemia cells

**DOI:** 10.1038/s41598-023-41230-1

**Published:** 2023-09-02

**Authors:** Ai Asai-Nishishita, Masahiro Kawahara, Goichi Tatsumi, Masaki Iwasa, Aya Fujishiro, Rie Nishimura, Hitoshi Minamiguchi, Katsuyuki Kito, Makoto Murata, Akira Andoh

**Affiliations:** 1 Division of Hematology, Department of Medicine, Shiga University of Medical Science, Seta-Tsukinowa, Otsu, Shiga 520-2192 Japan; 2 Department of Hematology and Oncology, Graduate School of Medicine, Kyoto University, 54 Shogoin-Kawahara-cho, Sakyo-ku, Kyoto, 606-8397 Japan; 3 Division of Gastroenterology, Department of Medicine, Shiga University of Medical Science, Seta-Tsukinowa, Otsu, Shiga 520-2192 Japan

**Keywords:** Haematological cancer, Cancer epigenetics, Molecular medicine

## Abstract

*FUS-ERG* is a chimeric gene with a poor prognosis, found in myelodysplastic syndromes (MDS) and acute myeloid leukaemia (AML). It remains unclear whether DNA hypomethylating agents, including azacitidine (Aza), are effective in FUS-ERG-harbouring AML and how FUS-ERG induces chemoresistance. Stable Ba/F3 transfectants with FUS-ERG were repeatedly exposed to Aza for 7 days of treatment and at 21-day intervals to investigate Aza sensitivity. Stable FUS-ERG transfectants acquired resistance acquired resistance after three courses of Aza exposure. RNA sequencing (RNA-seq) was performed when Aza susceptibility began to change; genes with altered expression or transcript variants were identified. Molecular signatures of these genes were analysed using gene ontology. RNA-seq analyses identified 74 upregulated and 320 downregulated genes involved in cell motility, cytokine production, and kinase activity. Additionally, 1321 genes with altered transcript variants were identified, revealing their involvement in chromatin organisation. In a clinical case of AML with FUS-ERG, we compared whole-genome alterations between the initial MDS diagnosis and AML recurrence after Aza treatment. Genes with non-synonymous or near mutations in transcription regulatory areas (TRAs), additionally detected in AML recurrence, were collated with the gene list from RNA-seq to identify genes involved in acquiring Aza resistance in the presence of FUS-ERG. Whole-genome sequencing of clinical specimens identified 29 genes with non-synonymous mutations, including BCOR, and 48 genes located within 20 kb of 54 TRA mutations in AML recurrence. These genes were involved in chromatin organisation and included NCOR2 as an overlapping gene with RNA-seq data. Transcription regulators involved in mutated TRAs were skewed and included RCOR1 in AML recurrence. We tested the efficacy of BH3 mimetics, including venetoclax and S63845, in primary Aza-resistant AML cells treated with FUS-ERG. Primary FUS-ERG-harbouring AML cells acquiring Aza resistance affected the myeloid cell leukaemia-1 (MCL1) inhibitor S63845 but not while using venetoclax, despite no mutations in *BCL2*. FUS-ERG promoted Aza resistance after several treatments. The disturbance of chromatin organisation might induce this by co-repressors, including BCOR, NCOR2, and RCOR1. MCL1 inhibition could partially overcome Aza resistance in FUS-ERG-harbouring AML cells.

## Introduction

*FUS-ERG* (also known as *TLS-ERG*) is a rare chimeric gene generated by t(16;21) (p11;q22) in acute myeloid leukaemia (AML) and myelodysplastic syndromes (MDS)^1^. AML or MDS with FUS-ERG has been reported to have a poor prognosis even with allogeneic haematopoietic stem cell transplantation^2,3^. FUS-ERG is neither specified in the latest World Health Organization classification nor listed as an adverse risk in the European LeukemiaNet classification^4,5^. However, it is listed as a poor prognostic chromosomal change in children with AML^6^.

The molecular mechanisms underlying the leukemogenesis of FUS-ERG have been investigated in several studies. For instance, FUS-ERG alters myeloid and erythroid differentiation and enhances the proliferative and self-renewal capacities of myeloid progenitor cells^7^; this is presumably linked to the dysfunction of ERG, which is a key transcription factor in the maintenance of haematopoietic stem cells and haematopoiesis of erythrocytes and megakaryocytes^8,9^. In addition, FUS-ERG reportedly colocalises with other key transcription factors in specific genomic areas responsible for transcriptional regulation and interferes with haematopoietic differentiation^10^. Furthermore, FUS-ERG binds to RNA polymerase II and interferes with RNA splicing^11^; this results from the dysregulation of FUS, which belongs to the FUS, EWSR1, and TAF15 family and is related to gene expression via transcription regulation and alternative splicing regulation^11,12^. However, the mechanisms underlying chemoresistance and poor prognosis in AML or MDS with FUS-ERG remain unclear.

DNA hypomethylating agents (HMA) , including azacitidine (Aza), have recently been used as first-line drugs in high-risk MDS and elderly patients or those with refractory AML^13,14^. Most studies have demonstrated that AML with FUS-ERG is refractory to traditional cytarabine-based regimens, but the efficacy of Aza has not been investigated. Although there is a case report that Aza is effective as a salvage therapy for paediatric AML with FUS-ERG and early relapse after allogeneic haematopoietic stem cell transplantation^15^, the direct effect of Aza on AML cells with FUS-ERG has not been demonstrated. In this study, we investigated whether Aza was effective against FUS-ERG.

## Methods

### Study overview

In this study conducted between January 2017 and October 2022, using haematopoietic cells stably expressing FUS-ERG, we investigated Aza sensitivity in cells with FUS-ERG and described gene expression profiles. Additionally, using samples from the clinical case patient, we compared whole-genome alterations between the initial diagnosis of MDS and the recurrence of AML after Aza treatment. We tested the efficacy of BH3 mimetics against primary FUS-ERG-bearing AML cells that acquired Aza resistance.

### Cells and constructs

To investigate the association between FUS-ERG and Aza sensitivity, we established haematopoietic cells stably expressing FUS-ERG (Additional file 1; Figure S1). Ba/F3 cells were authenticated using short tandem repeat analysis by the American Type Culture Collection (Manassas, VA, USA) and grown in Iscove's modified Dulbecco's medium (Nacalai, Kyoto, Japan) supplemented with 20% foetal bovine serum, 20% WEHI supernatant, and 1% penicillin/streptomycin in a humidified atmosphere containing 5% carbon dioxide at 37 °C. WEHI cells were cultured in high-glucose-containing Dulbecco's modified eagle medium (Nacalai) supplemented with 10% foetal bovine serum, 5 × 10^–5^ M β-mercaptoethanol, and 1% penicillin/streptomycin. The supernatant was collected after centrifugation and 0.22 µm filtration for cytokine supplementation. The full length of FUS-ERG was amplified from the cDNA of the patient sample using the primers GGTACTCAGCGGTGTTGGAA and CTTCCCCAGCCCCAGTAAAG and constructed into pcDNA3.1. cDNA was synthesised from the total RNA using SuperScript III Reverse Transcriptase (Invitrogen, Waltham, MA, USA). Total RNA was isolated using an RNeasy Mini Kit (Qiagen, Hilden, Germany). FUS-ERG-transduced Ba/F3 cells were established using Nucleofector by Amaxa (Lonza, Basel, Switzerland) and selected with 1 mg/ml G418 (Nacalai).

### Aza treatment assay

FUS-ERG- or empty vector-transduced Ba/F3 cells were treated with the indicated Aza concentration for seven days with 21-day intervals of Aza-free conditions in between. The Aza-containing medium was entirely or partially changed daily, and live cells were manually counted using light microscopy at 40 × after staining the preparation with trypan blue (Nacalai) on days two, four, and seven. The surviving cells in 0.5 µM Aza of the initial course and the second course and 1 µM of the third course were used for the second, third, and fourth course Aza treatment, respectively.

### RNA sequencing

Total RNA of FUS-ERG- or empty vector-transduced Ba/F3 cells was isolated before the fourth Aza treatment. The TruSeq stranded mRNA LT Sample Prep Kit (Illumina, San Diego, CA) was used to prepare the libraries. Paired-end RNA sequencing (RNA-seq) was performed using Novaseq6000 with 40–60 million reads per sample. The trimmed reads were mapped to the reference genome using HISAT2 (http://daehwankimlab.github.io/hisat2/). Transcripts were assembled using StringTie (http://ccb.jhu.edu/software/stringtie/index.shtml) with aligned reads. Finally, expression profiles were represented as read counts and normalisation values, which were calculated based on transcript length and depth of coverage. Normalisation values were provided as fragments per kilobase of transcript per million fragments mapped. Gene Ontology (GO) analysis was performed using Metascape (https://metascape.org/gp/index.html#/main/step1). The alignment analysis was performed using BLAST (https://blast.ncbi.nlm.nih.gov/Blast.cgi).

### Patient samples and whole-genome sequencing

This study was approved by the ethics committee of Shiga University of Medical Science (permission number G-150). Bone marrow mononuclear cells were purified using Ficoll-Paque (GE Healthcare, Chicago, IL, USA) from a patient initially diagnosed with MDS with FUS-ERG and relapsed as AML with FUS-ERG after obtaining written informed consent. Buccal swabs were used to subtract germline mutations. Genomic DNA extraction and library preparation were performed using a QIAmp DNA mini kit (Qiagen) and TruSeq Nano DNA Library Prep Kit (Illumina). Whole-genome sequencing (WGS) was performed using NovaSeq6000 (Illumina). Total reads were 1.2–1.5 billion per sample. Sequencing was performed by paired-end and multiplex methods using Illumina-specified flow cells with an average depth greater than 50 × . Uniformity (Pct > 0.2*mean) was over 97%. FASTQ files were mapped to the hg19 human reference genome using the DRAGEN: 3.9.5 software. The identification conditions of genome alterations were set at more than 20% of at least five reads after the subtraction of germline alterations. Genome areas involved in transcription regulators were visualised by reconstructing chromatin immunoprecipitation sequencing data from the ENCODE transcription factor binding site (TFBS) cluster (v3) dataset (wgEncodeRegTfbsClusteredV3.bed.gz) in the University of California Santa Cruz Genome Browser (https://genome.ucsc.edu/index.html). This study was approved by the ethics committee of our institution (permission number G-150).

### Drug sensitivity in primary AML cells

Primary AML cells from the patient were separated using Ficoll-Paque and maintained at 3 × 10^5^ cells/mL in Iscove's Modified Dulbecco's Medium supplemented with 20% foetal bovine serum, 1% penicillin/streptomycin, and 10 ng/mL human interleukin-3 in a humidified atmosphere containing 5% carbon dioxide at 37 °C. Primary AML cells were treated with the indicated Aza and venetoclax or Aza and S63845 concentrations for seven days. The drug-containing medium was partially changed daily, and live cells were counted as previously described after staining the preparation with trypan blue on day 7. Venetoclax and S63845 were administered to primary AML cells derived from the patient in combination with 2 µM Aza. For the analysis of drug sensitivity, cells were incubated with annexin-V BioLegend (San Diego, CA, USA) and 4′,6-diamidino-2-phenylindole (Nacalai) and analysed with BD LSRFortessa X-20 and Flowjo v10 software (BD Biosciences, San Jose, CA).

### Reagents

Where experiments called for their use, 5-Aza (Sigma-Aldrich, St. Louis, MO), venetoclax (Abcam, Cambridge, UK), and S63845 (Selleck, Houston, TX) were dissolved in dimethyl sulfoxide (DMSO).

### Statistical analysis

Analysis of variance was used for the comparative analysis of viability in the cell growth inhibition assay. Statistical significance was set at a *P* value < 0.05.

### Data sharing statement

RNA-seq data are available at GEO under accession number GSE217967. WGS genotype data have been deposited at the Japanese Genotype–phenotype Archive (JGA, https://www.ddbj.nig.ac.jp/jga), which is hosted by the Bioinformation and DDBJ Center, under accession number JGAS000587.

### Ethics approval statement

This study was approved by the ethics committee of Shiga University of Medical Science (permission number G-150). This study was performed in accordance with the Declaration of Helsinki**.**

### Patient consent statement

Patient's samples were collected after obtaining written informed consent for this study and publication.

## Results

### FUS-ERG could adversely affect Aza sensitivity through multiple exposures

**Figure 1:**
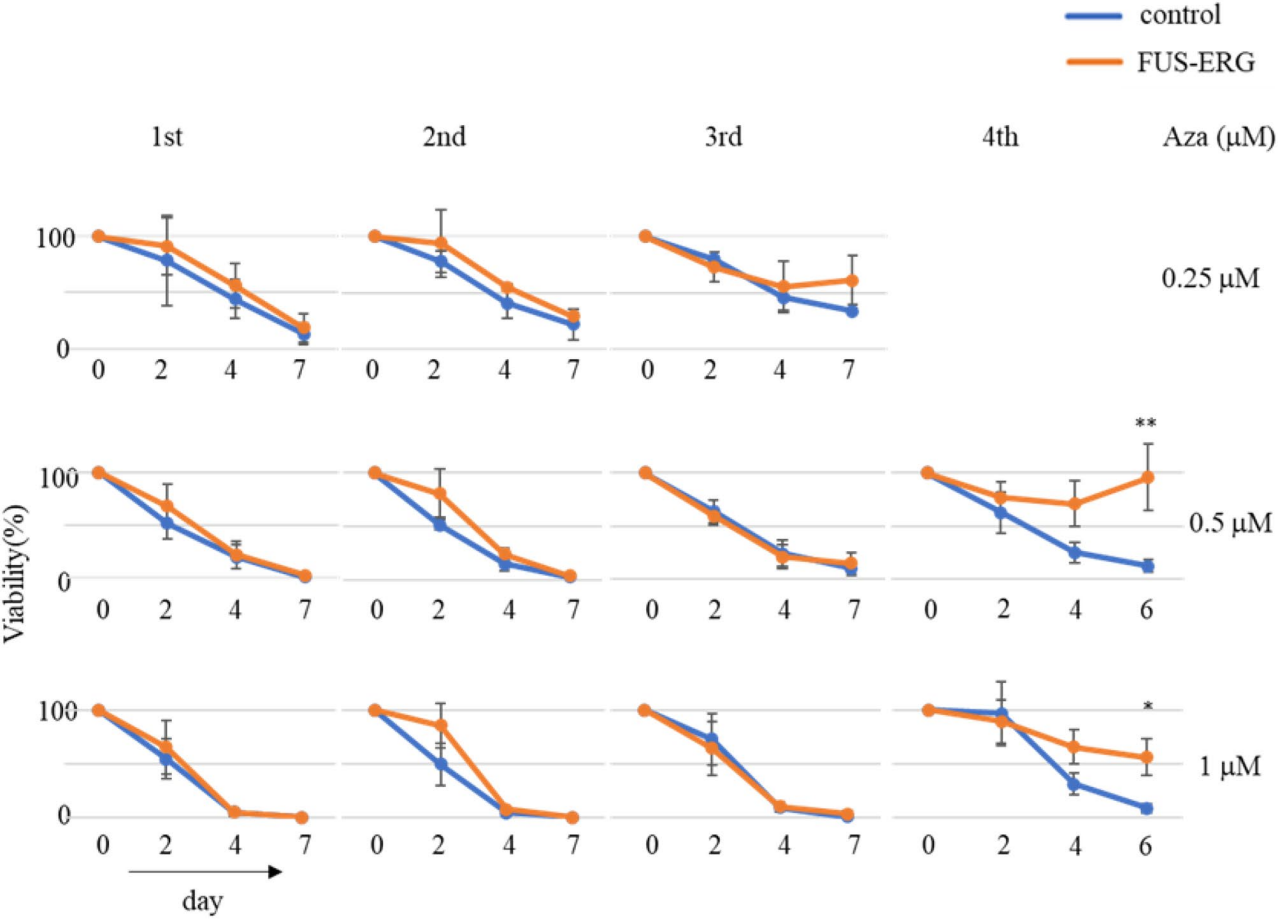
Viability of FUS-ERG expressed Ba/F3 cells in each repeated course of azacitidine treatment. The viability of each course of azacitidine treatment is presented. Cells that survived 0.5 µM or 1 µM in the previous course were used in the second and third courses or the fourth course, respectively. Viability (%) is compared with d 0 on the vertical axes. The number of courses and days after starting treatment are shown in the upper row and horizontal axes, respectively. Azacitidine concentration (µM) is shown in the right column. Experiments were independently performed three times. Mean and standard deviation are shown. Asterisks indicate *P* values (* < 0.05 and ** < 0.01).

Aza sensitivity was comparable between empty vector-transduced (hereafter referred to as control) and FUS-ERG cells in 0.25, 0.5, and 1  µM Aza conditions of the first course (Fig. 1). Thus, surviving cells in the 0.5 µM Aza condition were repopulated and re-treated after 21-day intervals of the Aza-free period. In the second course, Aza sensitivity was comparable between the control and FUS-ERG cells. In the 0.25 µM Aza condition of the third course, FUS-ERG cells exhibited a faint indication of Aza resistance, despite no apparent difference in the 0.5 and 1 µM Aza conditions. However, the cells that survived in the 1 µM Aza condition of the third course exhibited significant Aza resistance in the 0.5 and 1 µM Aza conditions of the fourth course, suggesting that FUS-ERG might contribute to the attenuation of Aza sensitivity through repeated exposures to Aza.

### Multiple Aza exposures may alter the signatures of cell motility, cytokine production, kinase activity, and chromatin organisation in FUS-ERG cells

**Figure 2:**
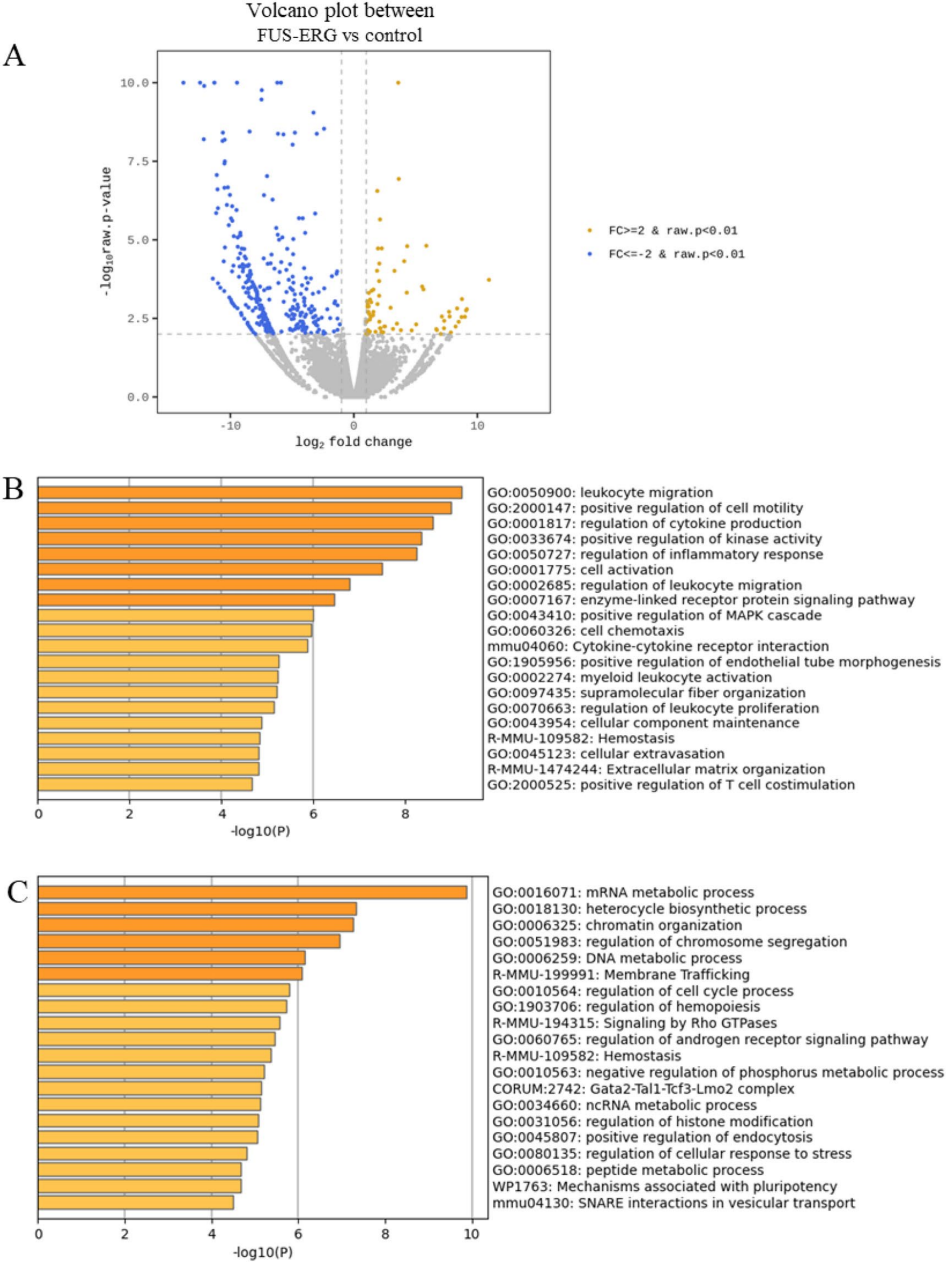
Gene expression profiling before the fourth azacitidine exposure. Total RNA purified from control cells and FUS-ERG-expressed cells was analysed. (**A**) Volcano plot of FUS-ERG vs. control with |Fold Change (FC)|≥ 2 and *P* < 0.01. (**B**) Top 20 Gene Ontology functional annotations for differentially expressed genes with |FC|≥ 2 and *P* < 0.01. (**C**) Schematic diagram of mouse NCOR2 domains and the location of amino acid sequence changes in transcript variants A and B using BLAST and UniProt (https://www.uniprot.org/uniprotkb/Q9WU42/entry). NCOR2 has three repression domains (RD1, RD2, and RD3), two SW13/ADA2/NCoR/TFIIB (SANT), and two nuclear ID1 and ID2. RD1 interacts with GPS2 and TBL1, and HDAC3 binds directly to SANT1, the deacetylase activation domain (DAD), and SANT2, a histone interaction domain that recognises histones in the context of other proteins. IDs bind to nuclear receptors. (**D**) Top 20 Gene Ontology functional annotations for genes with differentially expressed transcript variants with |FC|≥ 2 and *P* < 0.01. Splicing alternative transcript types are the containment of references and at least one junction match.

To understand the molecular mechanisms underlying the loss of Aza sensitivity in FUS-ERG cells exposed to multiple Aza, we performed RNA-seq of control and FUS-ERG cells just before the fourth course. A comparison of gene expression profiles identified 74 upregulated genes and 320 downregulated genes in FUS-ERG cells (Fig. 2A). GO analysis of the 74 upregulated genes indicated functional annotations associated with leukocyte migration and cellular extravasation (Additional file 1; Figure S2A). Moreover, GO analysis of the 320 downregulated genes indicated functional annotations associated with the regulation of cytokine production and cell motility (Additional file 1; Figure S2B). Recognizing that gene function is not solely inferred from upregulation or downregulation, we conducted GO analysis for differentially expressed genes with |Fold Change (FC)|≥ 2. This analysis disclosed functional annotations connected to the regulation of cell motility, cytokine production, inflammatory response, and kinase activity (Fig. 2B). Within cell motility, genes implicated in positive regulation were listed; however, their expression was largely downregulated (Additional file 1; Figure S2C). It has been reported that the HMA 5-aza-2'-deoxycytidine promotes the migration of leukaemia cells^16^. The observed downregulation of cell motility might be associated with resistance to HMA. In the context of cytokine production, genes such as *Csf1r* and *Adora2b*, involved in the positive regulation of chemokines, exhibited downregulation. Genes associated with the negative regulation of tumour necrosis factor (TNF) production, such as *Cx3cl1* and *Lbp*, were upregulated, whereas those linked to the positive regulation of TNF production such as *Fcer1g* and *Tlr1* were downregulated (Additional file 1; Figure S2D). It has been reported that 5-Aza-induced apoptosis possibly stems from induced expression of cytotoxic cytokines such as TNF-α^17^. Resistance to HMAs might be associated with the downregulation of cytotoxic cytokines. These distinct gene signatures that are seemingly downregulated could arise from the dysregulation of gene expression in FUS-ERG. In addition, we investigated alterations in transcript variants because FUS is a key molecule for RNA splicing. We identified 1321 genes with altered transcript variants. As molecular signatures directed by these genes, mRNA, DNA metabolic processes, and chromatin organisation were revealed in the high-rank GO annotations (Fig. 2C).

### Additional alterations in BCOR and a transcription regulatory area near NCOR2 may disturb chromatin organisation and lead to Aza resistance in FUS-ERG-harbouring AML cells

**Figure 3:**
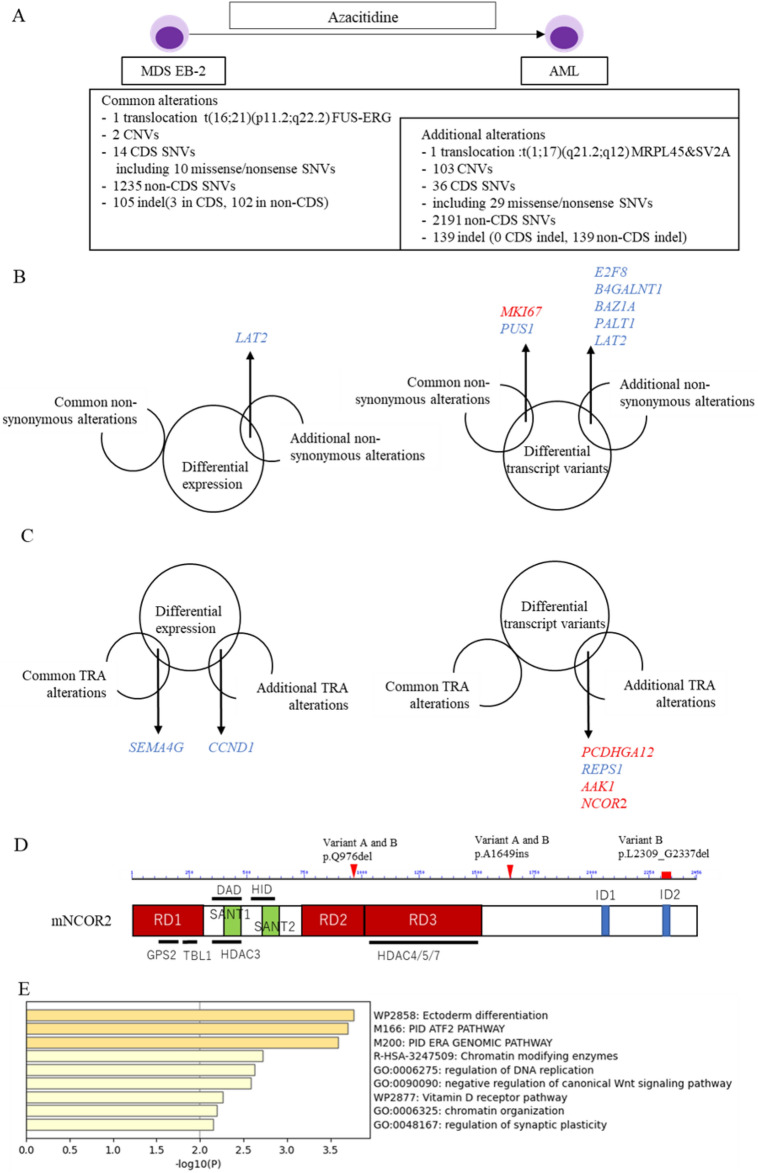
Whole-genome sequence of transformed AML case from MDS with FUS-ERG during azacitidine treatment. (**A**) Whole-genome sequence data for the initial diagnosis sample diagnosed as MDS and for the relapsed sample diagnosed as transformed AML. Common alterations mean alterations detected in both samples. Additional alterations mean alterations additionally detected only in transformed AML. (**B**) A Venn diagram illustrating the overlaps between genes exhibiting non-synonymous alterations and genes displaying differential expression or differential transcript variants in FUS-ERG Ba/F3 cells following multiple Aza exposures. Genes in red depict upregulated genes or genes with an increased number of altered transcript variants, while genes in blue represent downregulated genes or genes with a decreased number of altered transcript variants. (**C**) A Venn diagram demonstrating the overlaps between genes neighbouring additional TRA alterations and genes displaying differential expression or differential transcript variants in FUS-ERG Ba/F3 cells following multiple Aza exposures. (**D**) A schematic diagram showcasing mouse NCOR2 domains along with the positions of amino acid sequence changes in transcript variants A and B using BLAST and UniProt (https://www.uniprot.org/uniprotkb/Q9WU42/entry). NCOR2 encompasses three repression domains (RD1, RD2, and RD3), two SW13/ADA2/NCoR/TFIIB (SANT) domains, and two nuclear ID1 and ID2. RD1 interacts with GPS2 and TBL1, whereas HDAC3 directly binds to SANT1, the deacetylase activation domain (DAD), and SANT2, a histone interaction domain that recognises histones in the context of other proteins. IDs bind to nuclear receptors. (**E**) Gene Ontology functional annotations for the genes additional non-synonymous alterations or genes located near additional TRA alterations.

We recently encountered a patient with MDS with excess blast 2 harbouring t(16;21)(p11.2;q22.2), generating the FUS-ERG chimeric gene, and finally transformed to AML despite achieving complete remission once by Aza (Additional file 1; Figures S3 and S4). To investigate the genomic alterations in acquiring Aza resistance in the MDS clone with FUS-ERG, we performed WGS of BMMNCs (Fig. 3A). Common alterations in both initial MDS and relapse AML were a single translocation of t(16;21)(p11.2;q22.2), two copy number variants, and 14 single nucleotide variants (SNVs) in cording sites (CDS), comprising 10 non-synonymous SNVs, including *MICAL3, SH3BP4, MKI67, GABRR3, ZNF674, CCT8L2, PUS1, PLXNB3, LRIG2*, and *FRMD4A*; 1,235 SNVs in non-CDS; and 105 insertion/deletion (indel) including three indels in CDS, including *KRAS c.504delG* (p.L168fs)　(Additional file 1; Table S3). Additional alterations detected only in relapsed AML were a single translocation of t(1;17)(q21.2;q12), 103 copy number variants that were primarily located on chromosome 1q reflecting the karyotype, 36 SNVs in CDS including 29 non-synonymous SNVs including *BCOR c.3340G* > *T* (p.E1114*), 2,191 SNVs in non-CDS, and 139 indels including none in CDS (Additional file 1; Table S4). The association of these non-synonymous alterations with MDS and AML has rarely been reported, except for *BCOR*. Most genes identified in the gene expression profiles from RNA-seq of FUS-ERG Ba/F3 cells did not have non-synonymous alterations in the WGS of patients with MDS transformed to AML, except for *LAT2*. *LAT2* was downregulated in RNA-seq. *E2F8, B4GALNT1, BAZ1A, PALT1* and *LAT2* were listed among both genes with altered transcript variants from RNA-seq and genes with non-synonymous alterations in WGS (Fig. 3B). The transcription levels of these genes were decreased.

Next, we focused on the genomic areas involved in transcriptional regulation. Several transcriptional regulators, including transcription factors and epigenetic cofactors, accumulate in specific genomic areas to regulate the expression of neighbouring genes^18^. Because a recent report showed that such areas are highly and specifically hypermutated in lymphoma^19^, we investigated alterations in transcription regulatory areas (TRAs), defined as genomic areas where five or more transcription regulators can be recruited according to the available dataset of chromatin immunoprecipitation sequencing [ENCODE TFBS cluster (v3) data]. As a result, 32 common alterations and 54 additional alterations were located in the TRAs. Within 20 kb of common or additional TRA alterations, we found 32 genes, including *PHF20* and *SP100*, and 48 genes, including *NCOR2* and *CCND1* (Additional file 1; Tables S5 and S6). On searching for overlaps between genes near additional TRA alterations and genes with differential expression or differential transcript variants from the RNA-seq data of FUS-ERG Ba/F3 cells, we identified five genes, *CCND1*, *PCDHGA12*, *REPS1*, *AAK1*, and *NCOR2* (Fig. 3C). Focusing on *NCOR2*, which affects genes involved in AML onset, two *NCOR2* transcript variants (hereafter named variant A and variant B) were identified in FUS-ERG cells and did not completely match all reported isoforms according to the alignment analysis with BLAST (Additional file 1; Tables S1 and S2). Compared to isoform X40, which has the highest matching rate to current variants, variant A had p.Q976del in the repression domain 2 (RD2) and p.A1649ins (Fig. 3D). Variant B had a further deletion of p.L2309_G2337 in the nuclear receptor-interacting domain 2 (ID2). These genes may be candidates for Aza resistance in FUS-ERG AML cells. Furthermore, GO analysis revealed that genes with additional non-synonymous alterations or near additional TRA alterations are involved in the dysregulation of chromatin-modifying enzymes and chromatin organisation (Fig. 3E).

### TRAs associated with RCOR1, CHD2, PHF8, STAT1, GATA1, RXRA, KDM5B, and HDAC1 were selectively targeted during the Aza treatment

**Figure 4:**
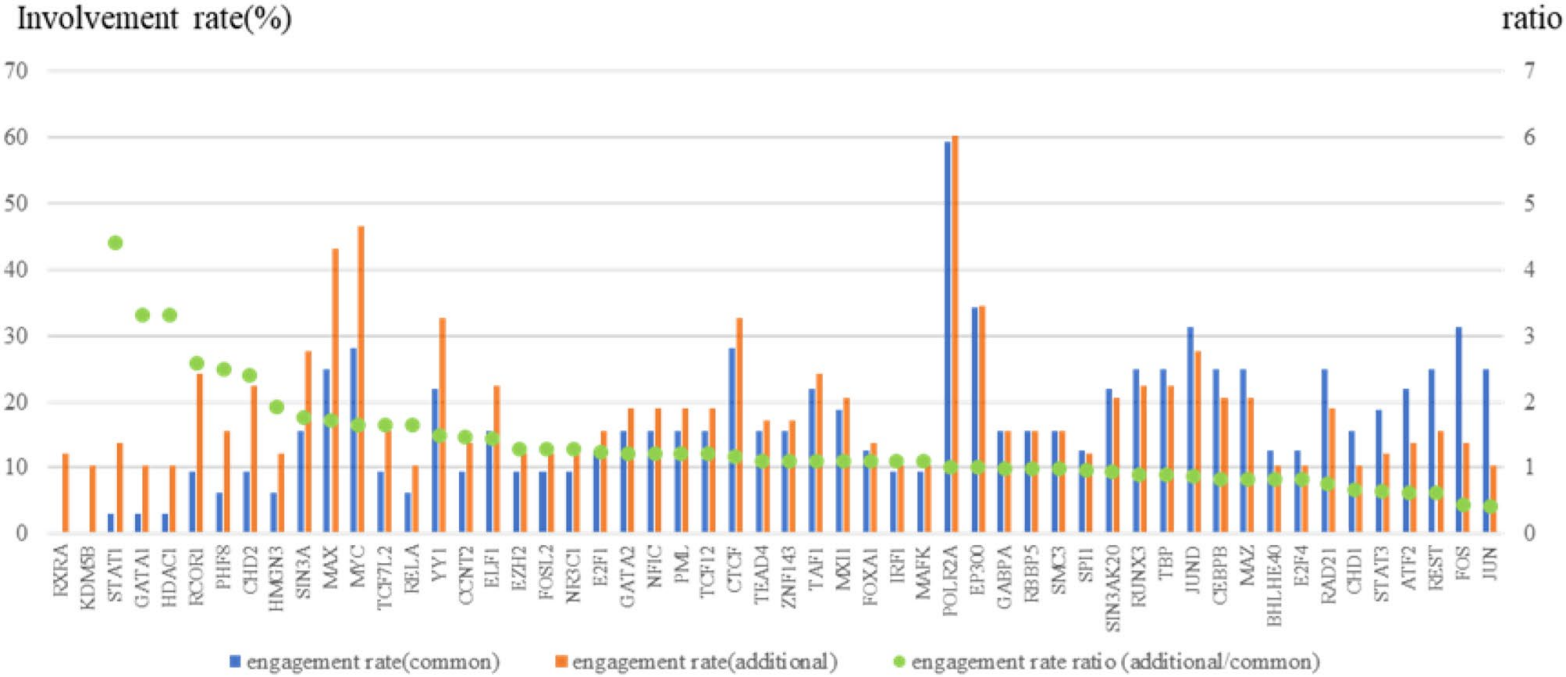
Involvement rate of each transcription regulator in TRA alterations. The involvement rate (%) shown in the left axis reveals the number of common TRA alterations (blue) or all additional TRA alterations (orange) involved in each transcription regulator, based on ENCODE TFBS cluster (v3) data. The ratio of involvement between common and additional TRA alterations in each transcriptional regulator is indicated by green circles and the right axis. Transcriptional regulators are listed on the horizontal axis in the order of the ratio of involvement.

We aggregated the extent to which each transcription regulator could be involved in the altered TRAs (Fig. 4). The involvement rate (%) revealed shared TRA alterations (illustrated by a blue bar) or additional TRA alterations (illustrated with an orange bar) involved in each transcription regulator. The ratio of involvement between common and additional TRA alterations in each transcriptional regulator is represented by green circles. TRAs involving POLR2A, EP300, and CTCF accounted for nearly 60%, 30%, and 30% respectively, of both common and additional TRA alterations; each ratio was 1, thereby signifying an equivalent degree of involvement. In the case of TRAs involving MYC, they constituted 28% of common TRA alterations and 47% of additional TRA alterations, resulting in a ratio of 1.65. These data suggest that TRAs involving specific transcription regulators might be selectively mutated in MDS with FUS-ERG. Moreover, RCOR1, CHD2, PHF8, STAT1, GATA1, and HDAC1 could be involved in more than two folds, similar to both additional and common TRA alterations. *RXRA* and *KDM5B* appeared to be exclusively engaged in additional TRA alterations, albeit less frequently. Finally, 29 genes, including *FBXL6*, *CDK11B*, *CCND1*, and *NCOR2*, were found within 20 kb of the additional TRA alterations involving these specific transcriptional regulators (Additional file 1; Table S6 and Figures. S5–8). These data suggest that TRA involving specific transcription regulators might be selectively targeted during Aza treatment in FUS-ERG-harbouring MDS clones.

### The myeloid cell leukaemia-1 (MCL1) inhibitor S63845 partially overcame Aza resistance in primary FUS-ERG-harbouring AML cells

**Figure 5:**
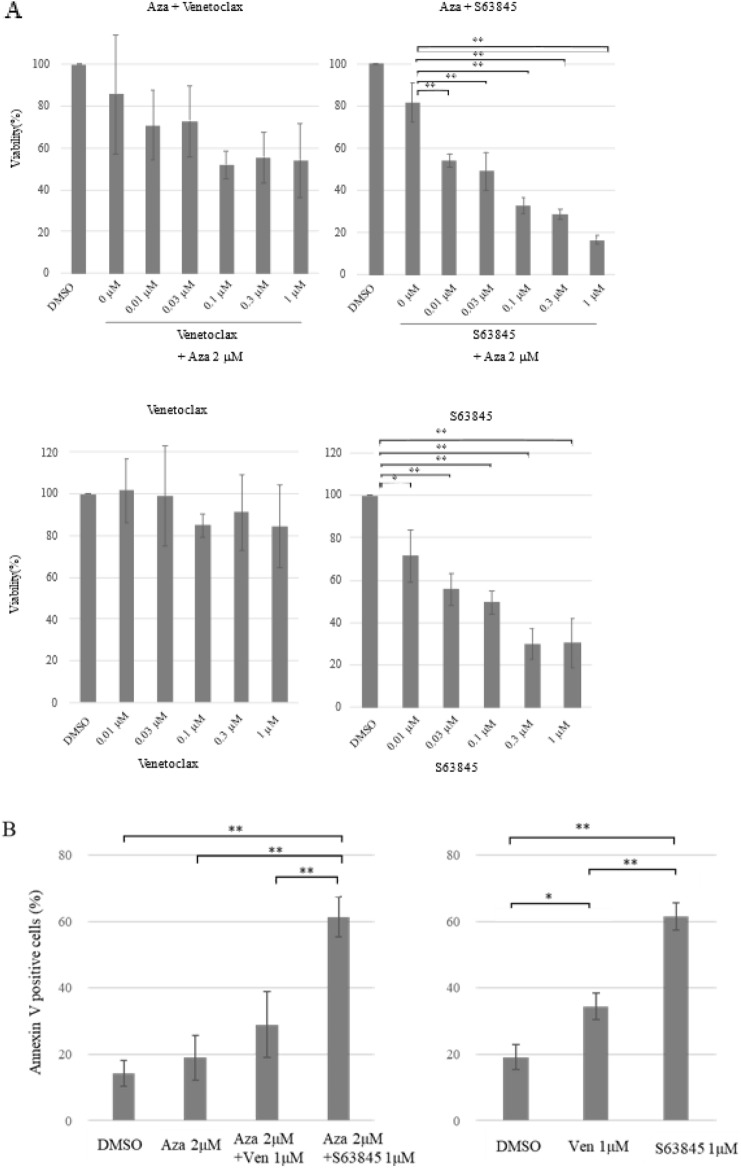
Susceptibility of AML primary cells to Aza plus venetoclax or S63845. (**A**) The viability of primary AML cells under various treatments involving venetoclax or S63845 alongside 2 µM Aza is depicted. Viability (%) is presented in comparison to DMSO by cell count after seven days of exposure on the vertical axis. Each drug concentration (µM) is shown on the horizontal axis. These experiments were performed independently three times. The mean and standard deviation are shown. Asterisks mean *P* value (* < 0.05 and ** < 0.01). (**B**) The ratio of AnnexinV-positive cells are presented under different conditions: no drugs (DMSO) and vehicle; 1 µM venetoclax; or 1 µM S63845, in combination with 2 µM Aza, as well as only 1 µM venetoclax or only 1 µM S63845 after 7 days of exposure. These experiments were performed independently three times. The mean and SD are shown. Asterisks mean *P* value (* < 0.05 and ** < 0.01).

Although we attempted to find effective drugs to overcome Aza resistance in AML with FUS-ERG, AML cells exhibited resistance, even with the addition of 1 µM venetoclax, a BCL2 inhibitor and the first-line drug for AML^20^ (Fig. 5A). Furthermore, the patient did not respond to all clinically available drugs, including venetoclax, despite no genomic alterations in the BCL2 gene body and its vicinity, based on our WGS data. As some reports suggest that AML cells resistant to the BCL-2 inhibitor are highly sensitive to S63845^21,22^, we attempted to treat AML cells with S63845. In contrast to venetoclax, the MCL1 inhibitor S63845^23^, which also holds promise as an AML therapeutic, exhibited efficacy even at a concentration as low as 0.01 μM, profoundly suppressing cell viability in a dose-dependent manner. No significant difference in efficacy was observed in combination with 2 μM Aza. However, following a 7-day treatment period, the co-administration of Aza and S63845 significantly increased the number of annexin-V-positive cells, in stark comparison to Aza treatment alone or in combination with venetoclax. Similar results were obtained with exclusive venetoclax or exclusive S63845 administration (Fig. 5B). These findings suggest that the MCL inhibitor S63845 exhibited a partial ability to overcome Aza resistance in AML with FUS-ERG.

## Discussion

Our findings demonstrated that FUS-ERG could not directly confer but did promote Aza resistance following multiple exposures. The development of resistance to HMA, despite their initial efficacy, is inevitable in many cases of AML and MDS. Gene mutations that predict Aza resistance have been vigorously explored, but none are conclusive^24^. In our clinical case, the *BCOR* level was altered following multiple courses of Aza. BCOR is an essential component of the polycomb repressive complex 1.1, which is recruited to unmethylated cytosine guanine dinucleotide islands via another component, KDM2B, to repress gene expression^25^ and is associated with the regulation of myeloid differentiation^26^. *BCOR* mutations are detected in approximately 4% of adult de novo AML, 8% of secondary AML, and 1.7% of paediatric AML cases^27–29^ and cause shorter transformation-free survival in MDS^30^. Recent reports have provided conflicting results regarding whether HMA resistance could be involved in *BCOR* mutation, together with or without mutations in other epigenetic modulators, including *ASXL1* and *RUNX1*^31,32^ No other additional mutations in our case were reported to be associated with HMA resistance. The *BCOR* mutation is involved not only in the transformation of MDS but also in Aza resistance.

Our WGS data revealed alterations in TRAs and specific genomic areas that multiple transcription regulators can bind. TRAs, including promoters and enhancers, influence genome structure and regulate gene expression^18^. Genes associated with TRA alterations commonly observed in initial MDS and relapsed AML, or those additionally observed only in relapsed AML, may be dysregulated and involved in developing MDS or acquiring Aza resistance, respectively. Furthermore, our RNA-seq data identified CCND1 genes with simply altered expression and *NCOR2* genes with isoform changes in FUS-ERG-transduced cells that acquired Aza resistance following multiple Aza exposures. Since FUS-ERG disturbs normal FUS function in RNA splicing regulation^11,12^, functional dysregulation by isoform change could be an alternative way to develop Aza-resistant clones in FUS-ERG-harbouring cells. As a result of the integrated analysis of WGS and RNA-seq data, four genes, including *NCOR2* (also known as *SMRT*), were identified as overlapping genes. NCOR2 is reported to be linked to leukemogenesis via functional dysregulation by major genetic alterations in AML, such as *FLT3* internal tandem duplication and *RUNX1-RUNX1T1* chimaera genes^33,34^. NCOR2 interacts with various nuclear receptors, including retinoic acid, and forms a complex with HDAC3 to repress its target genes^35,36^. Several NCOR2 isoforms have been reported, and aberrant splicing could lead to non-optimisation of the nuclear receptor corepressor family^37–39^. The current variant B has alterations in RD2 and ID2 and might, therefore, disturb NCOR2 function. The NCOR2/HDAC3 axis is critical for maintaining chromosomal structure and genomic stability^40^. The current GO analyses revealed chromatin organisation from RNA-seq data in FUS-ERG transfectants and WGS data in primary FUS-ERG-harbouring AML cells acquiring Aza resistance. Thus, genomic instability due to dysregulation of NCOR2 might cause the development of HMA-resistant clones in FUS-ERG-harbouring MDS and AML.

Our findings on TRA alterations also suggest that the target areas of several transcription regulators might be selectively altered. Alterations of TRAs that POLR2A and EP300 can recruit according to the available comprehensive ChIP-seq database were comparably observed in common alterations and additional alterations; this may be because FUS can bind to RNA polymerase II^11^ and regulate the histone acetyltransferase activity of EP300^41^. In contrast, TRAs involved in RXRA, KDM5B, STAT1, GATA1, HDAC1, RCOR1, PHF8, and CHD2 appeared to be selectively mutated in transformed AML cells during Aza treatment. KDM5B, HDAC1, and PHF8 are histone modifiers^42,43^, and RCOR1 cooperates with histone modifiers such as HDAC1^44^. CHD2, a modifier of chromatin structure, is reported to be somatically mutated in 8% of patients with chronic lymphocytic leukaemia^45^ and is less expressed in AML cells than in normal haematopoietic cells^46^. Considering recent reports that TRAs are hypermutated in haematological malignancies^19,47^, selective dysregulations of TRAs may be a part of the causes of HMA resistance.

Venetoclax, combined with Aza, currently used as a preferred regimen for AML^20^, had no in vitro effect on primary AML cells or clinical effect on our AML patient with FUS-ERG. This mechanism remains unclear because our WGS data showed no evidence of mutations in or around the gene body of BCL-2. In contrast, S63845, an MCL1 inhibitor, was partially effective in primary AML cells. S63845 reportedly induces apoptosis of AML cells, and AML cells resistant to the BCL-2 inhibitor Aza are highly sensitive to S63845^21,22^. Further studies with larger cohorts are required to confirm whether MCL inhibitors can overcome HMA resistance. In this study, we proposed drug selection and anticancer drug resistance mechanisms for AML with FUS-ERG, despite the rarity of cases, which makes conducting clinical research with a large number of cases difficult. However, whether it can be used for other leukaemias remains unknown because the analysis only included AML with FUS-ERG.

## Conclusions

In conclusion, our findings suggest that dysregulation of BCOR and NCOR2 could cause the development of HMA-resistant clones from MDS or AML cells with FUS-ERG. In addition, selective mutations in TRAs involved in specific transcription regulators, including RCOR1, may contribute to the acquisition of HMA resistance. As these co-repressors are essential for regulating gene expression, further coordinated studies of mutations, DNA methylation levels, and histone modifications in TRAs are required to understand HMA resistance mechanisms.

### Supplementary Information

Supplementary Information.

## Data Availability

RNA-seq data are available at GEO under accession number GSE217967. WGS genotype data have been deposited at the Japanese Genotype–phenotype Archive (JGA, https://www.ddbj.nig.ac.jp/jga), which is hosted by the Bioinformation and DDBJ Center, under accession number JGAS000587.
