# Supplementary material for: FUS-ERG induces late-onset azacitidine resistance in acute myeloid leukaemia cells
Source: Sci Rep. 2023 Sep 2;13:14454. doi: 10.1038/s41598-023-41230-1 (PMC10475016; doi:10.1038/s41598-023-41230-1)
Supplement: Supplementary file 1 — Supplementary Information. [file 41598_2023_41230_MOESM1_ESM.docx]

**FUS-ERG induces late-onset azacitidine resistance in acute myeloid leukaemia cells**

Ai Asai-Nishishita^1^, Masahiro Kawahara^1*^, Goichi Tatsumi^2^, Masaki Iwasa^1^, Aya Fujishiro^1^, Rie Nishimura^1^, Hitoshi Minamiguchi^1^, Katsuyuki Kito^1^, Makoto Murata^1^, and Akira Andoh^3*^

^1^Division of Hematology, Department of Medicine, Shiga University of Medical Science, Shiga, Japan

^2^Department of Hematology and Oncology, Graduate School of Medicine, Kyoto University, Kyoto, Japan

^3^Division of Gastroenterology, Department of Medicine, Shiga University of Medical Science, Shiga, Japan

*Corresponding authors: Masahiro Kawahara and Akira Andoh

E-mail: [mkawahar@belle.shiga-med.ac.jp](mailto:mkawahar@belle.shiga-med.ac.jp), andoh[@belle.shiga-med.ac.jp](mailto:@belle.shiga-med.ac.jp),

Address: Seta-Tsukinowa, Otsu, Shiga, 520-2192, Japan

TEL: +81-77-548-2217

FAX: +81-77-548-2219

**Supplementary Information**

**Supplementary** **materials and methods**

**Nested PCR and Sanger sequencing**

Total RNA was isolated using the RNeasy Mini Kit (Qiagen), and cDNA was synthesized using SuperScript III Reverse Transcriptase (Invitrogen, Waltham, MA, USA). Nested polymerase chain reaction (PCR) to detect the FUS-ERG chimeric gene was performed with Gflex (TAKARA, Shiga, Japan) and a 2720 Thermal Cycler (Applied Biosystems, Waltham, MA, USA). The first PCR was performed under the following conditions: 35 cycles of 98°C for 10 s, 63°C for 15 s, and 68°C for 30 s, with the following primers: AACTCCCCAGGGATATGGCT and GCGGCTGAGCTTATCGTAGT. One-twentieth of the first PCR product was used for the second PCR, of which the conditions were 35 cycles of 98°C for 10 s, 62°C for 15 s, and 68C for 20 s with primers GGTGGCTATGAACCCAGAGG and CCTCGTCGGGATCCGTCATC. The ethidium bromide staining confirmed the FUS-ERG fusion transcript on a 3% agarose gel. For Sanger sequencing to capture the fusion part, PCR products with primers CTATGGACAGCAGGACCGTG and CATAGTAGTAACGGAGGGCG were purified with the Wizerd SV Gel and PCR Clean-Up System (Promega, Madison, WI), and direct sequencing was performed.

**Western blotting**

Total cell lysates were prepared using RIPA buffer (10 mM Tris-HCl pH 7.5, 150 mM NaCl, 5 mM EDTA pH 8.0, 1% Triton X-100, 1% sodium deoxycholate, and 0.1% SDS) with protease inhibitor (Nacalai, Kyoto, Japan) and 100 mM PMSF. In addition, anti-ERG (rabbit mAb ab133264; Abcam, Cambridge, UK) and anti-β-actin (mouse mAb sc-47778; Santa Cruz Biotechnology, Dallas, TX) were used as primary antibodies, and ECL Anti-rabbit IgG-HRP (NA934vGE Healthcare, Little Chalfont, UK) and anti-mouse IgG-HRP (NA931vGE Healthcare) were used as secondary antibodies.

**Equipment and settings**

Chemiluminescence of western blotting was performed using Pierce ECL Western Blotting Substrate (Thermo Fisher Scientific, Waltham, MA). The developing device used CEPROS-SV (FUJIFILM, Tokyo, Japan) and was exposed for 1 minute. ATTO printgraph AE-6905H Image Saver HR (ATTO, Tokyo, Japan) was used for PCR gel photography. Microsoft Photo was used as the image processing software.

**Figure S1. FUS-ERG- or empty vector-transduced Ba/F3 cells.** (A) Western blotting. (A-1) Original blots. (B) Nested PCR. (B-1) Original gel. Lane 1, empty vector-transduced Ba/F3 cells (shown as the control in the manuscript); Lane 2, FUS-ERG-transduced Ba/F3 cells (shown as FUS-ERG in the manuscript). Lane *, positive control and Lane **, negative control.

**Figure S2. Gene expression profiling before the fourth azacitidine exposure.** (A) Top 20 Gene Ontology functional annotations for differentially expressed genes with FC ≥ 2 and *P* < 0.01. (B) Top 20 Gene Ontology functional annotations for differentially expressed genes with FC ≤ −2 and *P* < 0.01. (C) Changes in the expression of genes listed under the category of positive regulation of cell motility. (D) Changes in the expression of genes listed under the category of regulation of cytokine production.

**Figure S3.** **Myelodysplastic syndromes (MDS) with t(16;21)(p11.2;q22) case finally developing acute myeloid leukaemia (AML) during continuing azacitidine (Aza) treatment.** (A) Bone marrow smear and karyotype at the initial diagnosis as MDS. (B) Bone marrow smear and karyotype at relapse as AML. (C) Nested PCR of FUS-ERG. (C-1) Oliginal gel. Lane 1; initial diagnosis as MDS; Lane 2; after 10 courses of Aza treatment; Lane 3; relapse as AML. Lane *, positive control and Lane **, negative control.

**Figure S4. A direct sequence of BMMNCs at the onset of myelodysplastic syndrome (MDS) and during relapse as acute myeloid leukaemia (AML).** A vertical red line indicates the fusion point.

**Figure S5. View of an additional** **transcription regulatory area (TRA) alteration, chr8: 145582249 G>C.**

The top frame shows chromosome 8, and the red line indicates the position of the TRA alteration. The middle frame shows a close-up view near the TRA in Integrative Genomics Viewer (<https://software.broadinstitute.org/software/igv/>). The bottom frame shows a close-up view of the red box indicated in the middle frame and exhibits chromatin-precipitation sequencing data by reconstructing the ENCODE TFBS cluster (v3) data (wgEncodeRegTfbsClusteredV3.bed.gz) into the UCSC Genome Browser (<https://genome.ucsc.edu/index.html>). All data were analysed using the hg19 human reference genome data.

**Figure S6. View of an additional transcription regulatory area (TRA) alteration, chr1: 1590495 T>A.**

The top frame shows chromosome 1, and the red line indicates the position of the TRA alteration. The middle frame shows a close-up view near the TRA from the Integrative Genomics Viewer. The bottom frame shows a close-up view of the red box in the middle frame and exhibits chromatin-precipitation sequencing data by reconstructing the ENCODE TFBS cluster (v3) data (wgEncodeRegTfbsClusteredV3.bed.gz) into the UCSC Genome Browser. All data were analysed using the hg19 human reference genome data.

**Figure S7. View of an additional** **transcription regulatory area (TRA) alteration, chr11: 69459465 C>G.**

The top frame shows chromosome 11, and the red line indicates the position of the TRA alteration. The middle frame shows a close-up view near the TRA from the Integrative Genomics Viewer. The bottom frame shows a close-up view of the red box in the middle frame and exhibits chromatin-precipitation sequencing data by reconstructing the ENCODE TFBS cluster (v3) data (wgEncodeRegTfbsClusteredV3.bed.gz) into the University of California Santa Cruz Genome Browser. All data were analysed using the hg19 human reference genome data.

**Figure S8. View of an additional transcription regulatory area (TRA) alteration, chr12: 124882584 G>C.**

The top frame shows chromosome 12, and the red line indicates the position of the TRA alteration. The middle frame shows a close-up view near the TRA from the Integrative Genomics Viewer. The bottom frame shows a close-up view of the red box indicated in the middle frame and exhibits chromatin-precipitation sequencing data by reconstructing the ENCODE TFBS cluster (v3) data (wgEncodeRegTfbsClusteredV3.bed.gz) into the University of California Santa Cruz Genome Browser. All data were analysed using the hg19 human reference genome data.

**Figure S1**.


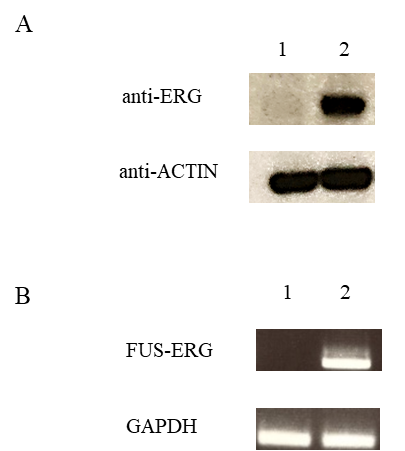


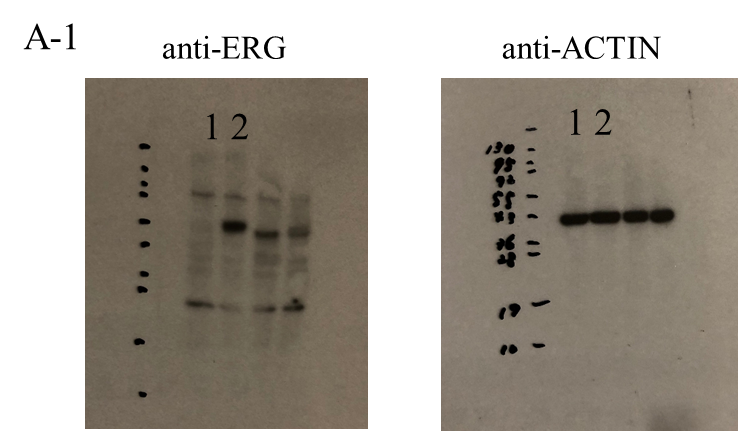


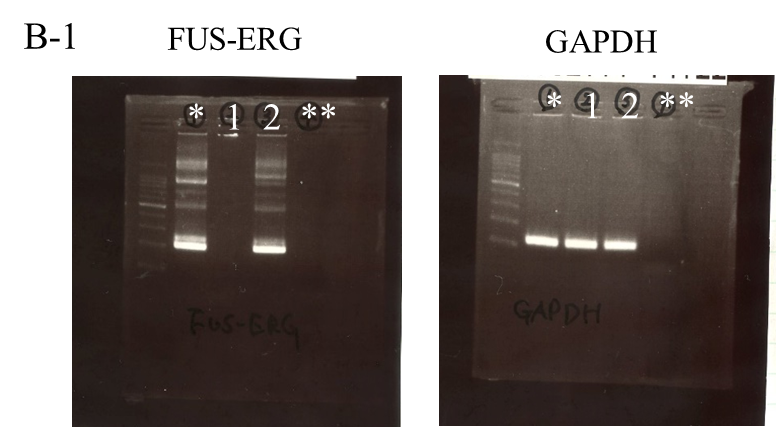


Figure S2

A

GO analysis of up-regulated genes


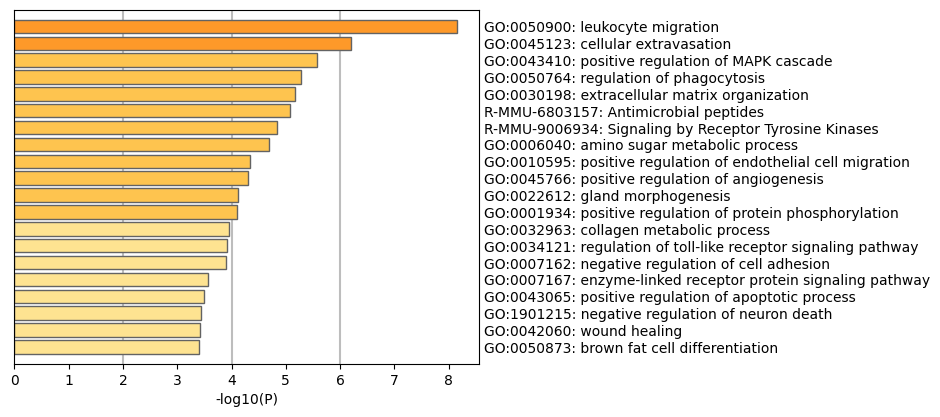


B
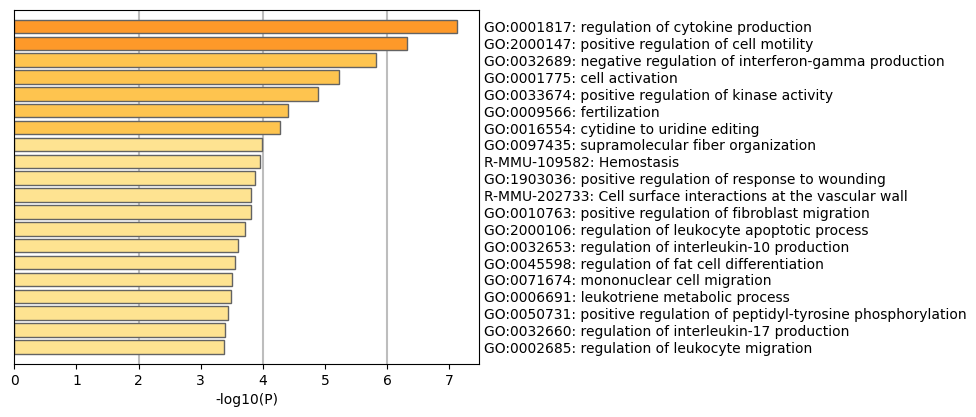


GO analysis of down-regulated genes

C.


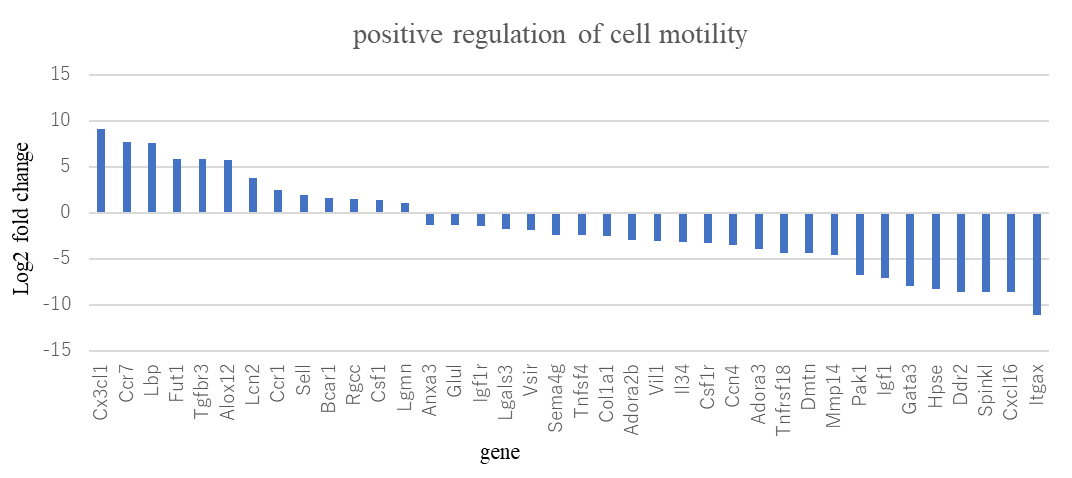


D.


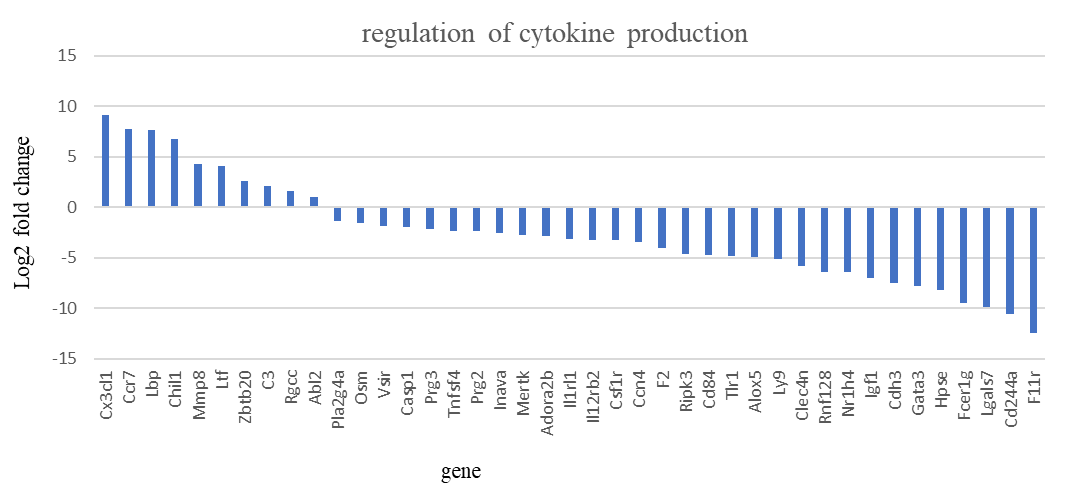


**Figure S3.**

**
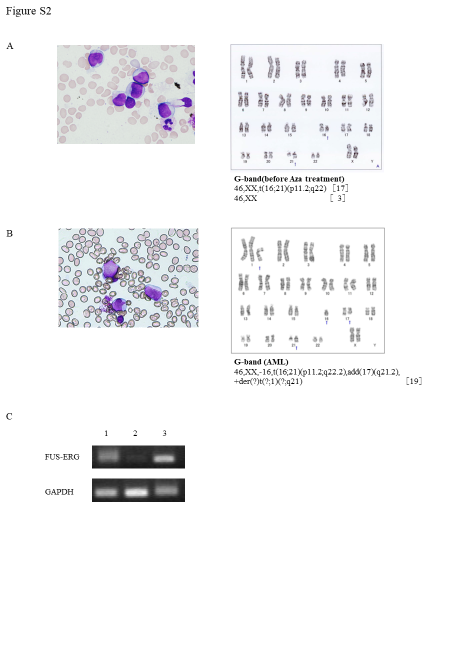
**

**
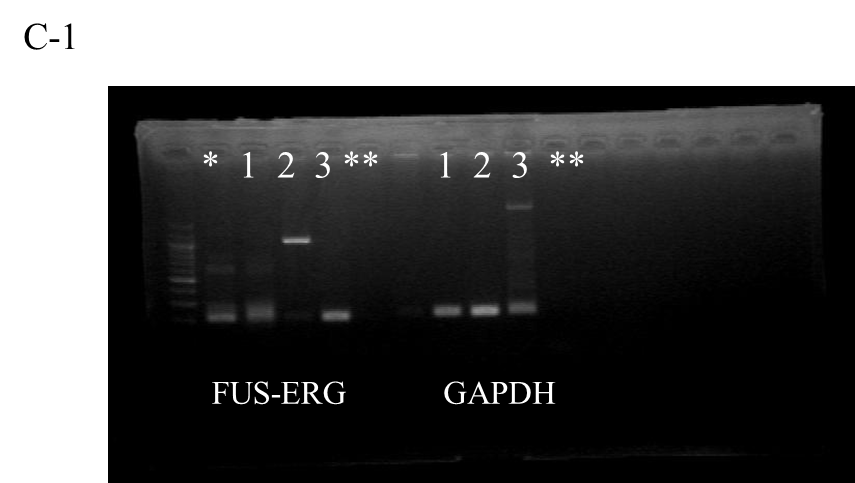
**

**Figure S4.**

**
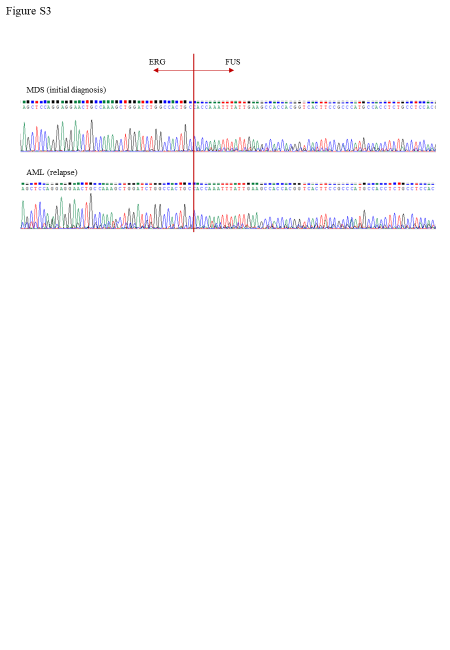
**

**Figure S5.**

**
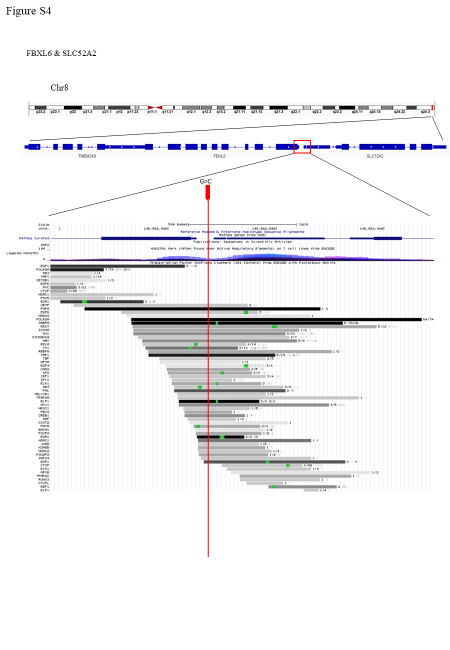
**

**Figure S6**

**
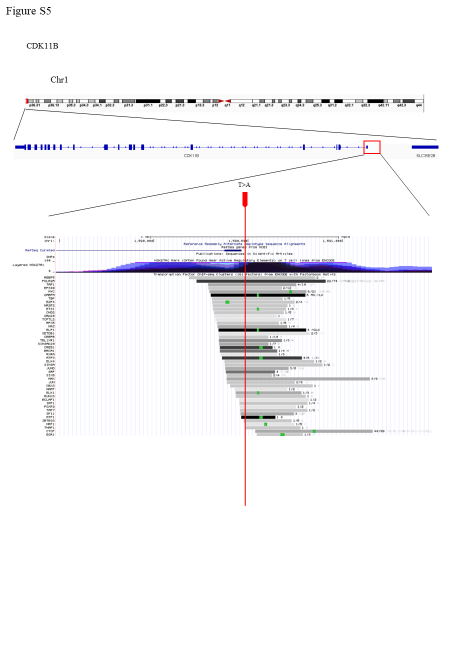
**

**Figure S7.**

**
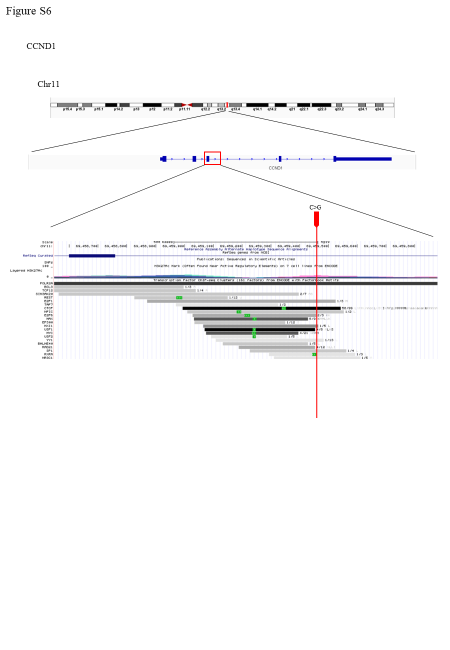
**

**Figure S8.**

**
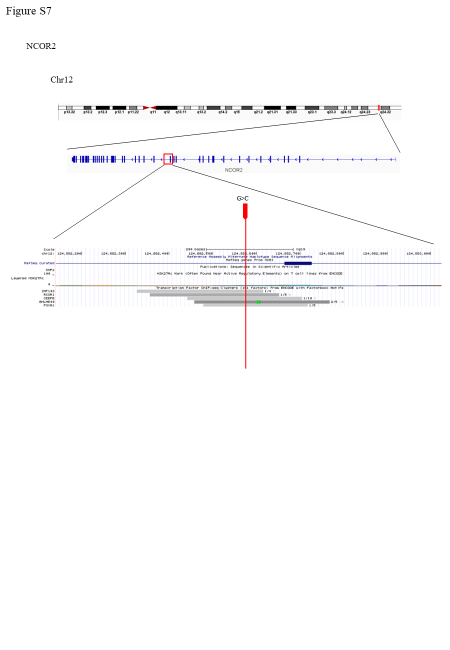
**

**Table S1.** Alignment of NCOR2 variant A at the top and NCOR2 isoform X40 [Mus musculus] at the bottom

XP_030110164.1 Length: 2456

1. 1 MSGSTQPVAQTWRAAEPRYPPHGISYPVQIARSHTDVGLLEYQHHPRDYTSHLSPGSIIQ

**X40.**1 MSGSTQPVAQTWRAAEPRYPPHGISYPVQIARSHTDVGLLEYQHHPRDYTSHLSPGSIIQ

61 PQRRRPSLLSEFQPGSERSQELHLRPESRTFLPELGKPDIEFTESKRPRLELLPDTLLRP

61 PQRRRPSLLSEFQPGSERSQELHLRPESRTFLPELGKPDIEFTESKRPRLELLPDTLLRP

121 SPLLATGQPSGSEDLTKDRSLAGKLEPVSPPSPPHADPELELAPSRLSKEELIQNMDRVD

121 SPLLATGQPSGSEDLTKDRSLAGKLEPVSPPSPPHADPELELAPSRLSKEELIQNMDRVD

181 REITMVEQQISKLKKKQQQLEEEAAKPPEPEKPVSPPPIESKHRSLVQIIYDENRKKAEA

181 REITMVEQQISKLKKKQQQLEEEAAKPPEPEKPVSPPPIESKHRSLVQIIYDENRKKAEA

241 AHRILEGLGPQVELPLYNQPSDTRQYHENIKINQAMRKKLILYFKRRNHARKQWEQRFCQ

241 AHRILEGLGPQVELPLYNQPSDTRQYHENIKINQAMRKKLILYFKRRNHARKQWEQRFCQ

301 RYDQLMEAWEKKVERIENNPRRRAKESKVREYYEKQFPEIRKQRELQERMQSRVGQRGSG

301 RYDQLMEAWEKKVERIENNPRRRAKESKVREYYEKQFPEIRKQRELQERMQSRVGQRGSG

361 LSMSAARSEHEVSEIIDGLSEQENLEKQMRQLAVIPPMLYDADQQRIKFINMNGLMDDPM

361 LSMSAARSEHEVSEIIDGLSEQENLEKQMRQLAVIPPMLYDADQQRIKFINMNGLMDDPM

421 KVYKDRQVTNMWSEQERDTFREKFMQHPKNFGLIASFLERKTVAECVLYYYLTKKNENYK

421 KVYKDRQVTNMWSEQERDTFREKFMQHPKNFGLIASFLERKTVAECVLYYYLTKKNENYK

481 SLVRRSYRRRGKSQQQQQQQQQQQQQQMARSSQEEKEEKEKEKEADKEEEKQDAENEKEE

481 SLVRRSYRRRGKSQQQQQQQQQQQQQQMARSSQEEKEEKEKEKEADKEEEKQDAENEKEE

541 LSKEKTDDTSGEDNDEKEAVASKGRKTANSQGRRKGRITRSMANEANHEETATPQQSSEL

541 LSKEKTDDTSGEDNDEKEAVASKGRKTANSQGRRKGRITRSMANEANHEETATPQQSSEL

601 ASMEMNESSRWTEEEMETAKKGLLEHGRNWSAIARMVGSKTVSQCKNFYFNYKKRQNLDE

601 ASMEMNESSRWTEEEMETAKKGLLEHGRNWSAIARMVGSKTVSQCKNFYFNYKKRQNLDE

661 ILQQHKLKMEKERNARRKKKKTPAAASEETAFPPAAEDEEMEASGASANEEELAEEAEAS

661 ILQQHKLKMEKERNARRKKKKTPAAASEETAFPPAAEDEEMEASGASANEEELAEEAEAS

721 QASGNEVPRVGECSGPAAVNNSSDTESVPSPRSEATKDTGPKPTGTEALPAATQPPVPPP

721 QASGNEVPRVGECSGPAAVNNSSDTESVPSPRSEATKDTGPKPTGTEALPAATQPPVPPP

781 EEPAVAPAEPSPVPDASGPPSPEPSPSPAAPPATVDKDEQEAPAAPAPQTEDAKEQKSEA

781 EEPAVAPAEPSPVPDASGPPSPEPSPSPAAPPATVDKDEQEAPAAPAPQTEDAKEQKSEA

841 EEIDVGKPEEPEASEEPPESVKSDHKEETEEEPEDKAKGTEAIETVSEAPLKVEEAGSKA

841 EEIDVGKPEEPEASEEPPESVKSDHKEETEEEPEDKAKGTEAIETVSEAPLKVEEAGSKA

901 AVTKGSSSGATQDSDSSATCSADEVDEPEGGDKGRLLSPRPSLLTPAGDPRASTSPQKPL

901 AVTKGSSSGATQDSDSSATCSADEVDEPEGGDKGRLLSPRPSLLTPAGDPRASTSPQKPL

961 DLKQLKQRAAAIPPI-VTKVHEPPREDTVPPKPVPPVPPPTQHLQPEGDVSQQSGGSPRG

961 DLKQLKQRAAAIPPIQVTKVHEPPREDTVPPKPVPPVPPPTQHLQPEGDVSQQSGGSPRG

1020 KSRSPVPPAEKEAEKPAFFPAFPTEGPKLPTEPPRWSSGLPFPIPPREVIKTSPHAADPS

1021 KSRSPVPPAEKEAEKPAFFPAFPTEGPKLPTEPPRWSSGLPFPIPPREVIKTSPHAADPS

1080 AFSYTPPGHPLPLGLHDSARPVLPRPPISNPPPLISSAKHPGVLERQLGAISQGMSVQLR

1081 AFSYTPPGHPLPLGLHDSARPVLPRPPISNPPPLISSAKHPGVLERQLGAISQGMSVQLR

1140 VPHSEHAKAPMGPLTMGLPLAVDPKKLAPFSGVKQEQLSPRGQAGPPESLGVPTAQETSV

1141 VPHSEHAKAPMGPLTMGLPLAVDPKKLAPFSGVKQEQLSPRGQAGPPESLGVPTAQETSV

1200 LRGTALGSATSGSITKGLPSTRAADGPSYRGSITHGTPADVLYKGTISRIVGEDSPSRLD

1201 LRGTALGSATSGSITKGLPSTRAADGPSYRGSITHGTPADVLYKGTISRIVGEDSPSRLD

1260 RAREDTLPKGHVIYEGKKGHVLSYEGGMSVSQCSKEDGRSSSGPPHETAAPKRTYDMMEG

1261 RAREDTLPKGHVIYEGKKGHVLSYEGGMSVSQCSKEDGRSSSGPPHETAAPKRTYDMMEG

1320 RVGRTVTSASIEGLMGRAIPEQHSPHLKEQHHIRGSITQGIPRSYVEAQEDYLRREAKLL

1321 RVGRTVTSASIEGLMGRAIPEQHSPHLKEQHHIRGSITQGIPRSYVEAQEDYLRREAKLL

1380 KREGTPPPPPPPRDLTETYKPRPLDPLGPLKLKPTHEGVVATVKEAGRSIHEIPREELRR

1381 KREGTPPPPPPPRDLTETYKPRPLDPLGPLKLKPTHEGVVATVKEAGRSIHEIPREELRR

1440 TPELPLAPRPLKEGSITQGTPLKYDSGAPSTGTKKHDVRSIIGSPGRPFPALHPLDIMAD

1441 TPELPLAPRPLKEGSITQGTPLKYDSGAPSTGTKKHDVRSIIGSPGRPFPALHPLDIMAD

1500 ARALERACYEESLKSRSGTSSGAGGSITRGAPVVVPELGKPRQSPLTYEDHGAPFTSHLP

1501 ARALERACYEESLKSRSGTSSGAGGSITRGAPVVVPELGKPRQSPLTYEDHGAPFTSHLP

1560 RGSPVTTREPTPRLQEGSLLSSKASQDRKLTSTPREIAKSPHSTVPEHHPHPISPYEHLL

1561 RGSPVTTREPTPRLQEGSLLSSKASQDRKLTSTPREIAKSPHSTVPEHHPHPISPYEHLL

1620 RGVTGVDLYRGHIPLAFDPTSIPRGIPLEAAAAAYYLPRHLAPSPTYPHLYPPYLIRGYP

1621 RGVTGVDLYRGHIPLAFDPTSIPRGIPLE-AAAAYYLPRHLAPSPTYPHLYPPYLIRGYP

1680 DTAALENRQTIINDYITSQQMHHNAASAMAQRADMLRGLSPRESSLALNYAAGPRGIIDL

1680 DTAALENRQTIINDYITSQQMHHNAASAMAQRADMLRGLSPRESSLALNYAAGPRGIIDL

1740 SQVPHLPVLVPPTPGTPATAIDRLAYLPTAPPPFSSRHSSSPLSPGGPTHLAKPTATSSS

1740 SQVPHLPVLVPPTPGTPATAIDRLAYLPTAPPPFSSRHSSSPLSPGGPTHLAKPTATSSS

1800 ERERERERERDKSILTSTTTVEHAPIWRPGTEQSSGAGGSSRPASHTHQHSPISPRTQDA

1800 ERERERERERDKSILTSTTTVEHAPIWRPGTEQSSGAGGSSRPASHTHQHSPISPRTQDA

1860 LQQRPSVLHNTSMKGVVTSVEPGTPTVLRSTSTSSPVRPAATFPPATHCPLGGTLEGVYP

1860 LQQRPSVLHNTSMKGVVTSVEPGTPTVLRSTSTSSPVRPAATFPPATHCPLGGTLEGVYP

1920 TLMEPVLLPKETSRVARPERPRVDAGHAFLTKPPAREPASSPSKSSEPRSLAPPSSSHTA

1920 TLMEPVLLPKETSRVARPERPRVDAGHAFLTKPPAREPASSPSKSSEPRSLAPPSSSHTA

1980 IARTPAKNLAPHHASPDPPAPTSASDLHREKTQSKPFSIQELELRSLGYHSGAGYSPDGV

1980 IARTPAKNLAPHHASPDPPAPTSASDLHREKTQSKPFSIQELELRSLGYHSGAGYSPDGV

2040 EPISPVSSPSLTHDKGLSKPLEELEKSHLEGELRHKQPGPMKLSAEAAHLPHLRPLPESQ

2040 EPISPVSSPSLTHDKGLSKPLEELEKSHLEGELRHKQPGPMKLSAEAAHLPHLRPLPESQ

2100 PSSSPLLQTAPGIKGHQRVVTLAQHISEVITQDYTRHHPQQLSGPLPAPLYSFPGASCPV

2100 PSSSPLLQTAPGIKGHQRVVTLAQHISEVITQDYTRHHPQQLSGPLPAPLYSFPGASCPV

2160 LDLRRPPSDLYLPPPDHGTPARGSPHSEGGKRSPEPSKTSVLGSSEDAIEPVSPPEGMTE

2160 LDLRRPPSDLYLPPPDHGTPARGSPHSEGGKRSPEPSKTSVLGSSEDAIEPVSPPEGMTE

2220 PGHARSTAYPLLYRDGEQGEPRMGSKSPGNTSQPPAFFSKLTESNSAMVKSKKQEINKKL

2220 PGHARSTAYPLLYRDGEQGEPRMGSKSPGNTSQPPAFFSKLTESNSAMVKSKKQEINKKL

2280 NTHNRNEPEYNIGQPGTEIFNMPAITGAGLMTCRSQAVQEHASTNMGLEAIIRKALMGGG

2280 NTHNRNEPEYNIGQPGTEIFNMPAITGAGLMTCRSQAVQEHASTNMGLEAIIRKALMGGG

2340 GKAKVSGRPSSRKAKSPAPGLASGDRPPSVSSVHSEGDCNRRTPLTNRVWEDRPSSAGST

2340 GKAKVSGRPSSRKAKSPAPGLASGDRPPSVSSVHSEGDCNRRTPLTNRVWEDRPSSAGST

2400 PFPYNPLIMRLQAGVMASPPPPGLAAGSGPLAGPHHAWDEEPKPLLCSQYETLSDSE 2456

2400 PFPYNPLIMRLQAGVMASPPPPGLAAGSGPLAGPHHAWDEEPKPLLCSQYETLSDSE 2456

**Table S2.** Alignment of NCOR2 variant B at the top and NCOR2 isoform X40 [Mus musculus] at the bottom

XP_030110164.1 Length: 2456

**B.** 1 MSGSTQPVAQTWRAAEPRYPPHGISYPVQIARSHTDVGLLEYQHHPRDYTSHLSPGSIIQ

**X40.**1 MSGSTQPVAQTWRAAEPRYPPHGISYPVQIARSHTDVGLLEYQHHPRDYTSHLSPGSIIQ

61 PQRRRPSLLSEFQPGSERSQELHLRPESRTFLPELGKPDIEFTESKRPRLELLPDTLLRP

61 PQRRRPSLLSEFQPGSERSQELHLRPESRTFLPELGKPDIEFTESKRPRLELLPDTLLRP

121 SPLLATGQPSGSEDLTKDRSLAGKLEPVSPPSPPHADPELELAPSRLSKEELIQNMDRVD

121 SPLLATGQPSGSEDLTKDRSLAGKLEPVSPPSPPHADPELELAPSRLSKEELIQNMDRVD

181 REITMVEQQISKLKKKQQQLEEEAAKPPEPEKPVSPPPIESKHRSLVQIIYDENRKKAEA

181 REITMVEQQISKLKKKQQQLEEEAAKPPEPEKPVSPPPIESKHRSLVQIIYDENRKKAEA

241 AHRILEGLGPQVELPLYNQPSDTRQYHENIKINQAMRKKLILYFKRRNHARKQWEQRFCQ

241 AHRILEGLGPQVELPLYNQPSDTRQYHENIKINQAMRKKLILYFKRRNHARKQWEQRFCQ

301 RYDQLMEAWEKKVERIENNPRRRAKESKVREYYEKQFPEIRKQRELQERMQSRVGQRGSG

301 RYDQLMEAWEKKVERIENNPRRRAKESKVREYYEKQFPEIRKQRELQERMQSRVGQRGSG

361 LSMSAARSEHEVSEIIDGLSEQENLEKQMRQLAVIPPMLYDADQQRIKFINMNGLMDDPM

361 LSMSAARSEHEVSEIIDGLSEQENLEKQMRQLAVIPPMLYDADQQRIKFINMNGLMDDPM

421 KVYKDRQVTNMWSEQERDTFREKFMQHPKNFGLIASFLERKTVAECVLYYYLTKKNENYK

421 KVYKDRQVTNMWSEQERDTFREKFMQHPKNFGLIASFLERKTVAECVLYYYLTKKNENYK

481 SLVRRSYRRRGKSQQQQQQQQQQQQQQMARSSQEEKEEKEKEKEADKEEEKQDAENEKEE

481 SLVRRSYRRRGKSQQQQQQQQQQQQQQMARSSQEEKEEKEKEKEADKEEEKQDAENEKEE

541 LSKEKTDDTSGEDNDEKEAVASKGRKTANSQGRRKGRITRSMANEANHEETATPQQSSEL

541 LSKEKTDDTSGEDNDEKEAVASKGRKTANSQGRRKGRITRSMANEANHEETATPQQSSEL

601 ASMEMNESSRWTEEEMETAKKGLLEHGRNWSAIARMVGSKTVSQCKNFYFNYKKRQNLDE

601 ASMEMNESSRWTEEEMETAKKGLLEHGRNWSAIARMVGSKTVSQCKNFYFNYKKRQNLDE

661 ILQQHKLKMEKERNARRKKKKTPAAASEETAFPPAAEDEEMEASGASANEEELAEEAEAS

661 ILQQHKLKMEKERNARRKKKKTPAAASEETAFPPAAEDEEMEASGASANEEELAEEAEAS

721 QASGNEVPRVGECSGPAAVNNSSDTESVPSPRSEATKDTGPKPTGTEALPAATQPPVPPP

721 QASGNEVPRVGECSGPAAVNNSSDTESVPSPRSEATKDTGPKPTGTEALPAATQPPVPPP

781 EEPAVAPAEPSPVPDASGPPSPEPSPSPAAPPATVDKDEQEAPAAPAPQTEDAKEQKSEA

781 EEPAVAPAEPSPVPDASGPPSPEPSPSPAAPPATVDKDEQEAPAAPAPQTEDAKEQKSEA

841 EEIDVGKPEEPEASEEPPESVKSDHKEETEEEPEDKAKGTEAIETVSEAPLKVEEAGSKA

841 EEIDVGKPEEPEASEEPPESVKSDHKEETEEEPEDKAKGTEAIETVSEAPLKVEEAGSKA

901 AVTKGSSSGATQDSDSSATCSADEVDEPEGGDKGRLLSPRPSLLTPAGDPRASTSPQKPL

901 AVTKGSSSGATQDSDSSATCSADEVDEPEGGDKGRLLSPRPSLLTPAGDPRASTSPQKPL

961 DLKQLKQRAAAIPPI-VTKVHEPPREDTVPPKPVPPVPPPTQHLQPEGDVSQQSGGSPRG

961 DLKQLKQRAAAIPPIQVTKVHEPPREDTVPPKPVPPVPPPTQHLQPEGDVSQQSGGSPRG

1020 KSRSPVPPAEKEAEKPAFFPAFPTEGPKLPTEPPRWSSGLPFPIPPREVIKTSPHAADPS

1021 KSRSPVPPAEKEAEKPAFFPAFPTEGPKLPTEPPRWSSGLPFPIPPREVIKTSPHAADPS

1080 AFSYTPPGHPLPLGLHDSARPVLPRPPISNPPPLISSAKHPGVLERQLGAISQGMSVQLR

1081 AFSYTPPGHPLPLGLHDSARPVLPRPPISNPPPLISSAKHPGVLERQLGAISQGMSVQLR

1140 VPHSEHAKAPMGPLTMGLPLAVDPKKLAPFSGVKQEQLSPRGQAGPPESLGVPTAQETSV

1141 VPHSEHAKAPMGPLTMGLPLAVDPKKLAPFSGVKQEQLSPRGQAGPPESLGVPTAQETSV

1200 LRGTALGSATSGSITKGLPSTRAADGPSYRGSITHGTPADVLYKGTISRIVGEDSPSRLD

1201 LRGTALGSATSGSITKGLPSTRAADGPSYRGSITHGTPADVLYKGTISRIVGEDSPSRLD

1260 RAREDTLPKGHVIYEGKKGHVLSYEGGMSVSQCSKEDGRSSSGPPHETAAPKRTYDMMEG

1261 RAREDTLPKGHVIYEGKKGHVLSYEGGMSVSQCSKEDGRSSSGPPHETAAPKRTYDMMEG

1320 RVGRTVTSASIEGLMGRAIPEQHSPHLKEQHHIRGSITQGIPRSYVEAQEDYLRREAKLL

1321 RVGRTVTSASIEGLMGRAIPEQHSPHLKEQHHIRGSITQGIPRSYVEAQEDYLRREAKLL

1380 KREGTPPPPPPPRDLTETYKPRPLDPLGPLKLKPTHEGVVATVKEAGRSIHEIPREELRR

1381 KREGTPPPPPPPRDLTETYKPRPLDPLGPLKLKPTHEGVVATVKEAGRSIHEIPREELRR

1440 TPELPLAPRPLKEGSITQGTPLKYDSGAPSTGTKKHDVRSIIGSPGRPFPALHPLDIMAD

1441 TPELPLAPRPLKEGSITQGTPLKYDSGAPSTGTKKHDVRSIIGSPGRPFPALHPLDIMAD

1500 ARALERACYEESLKSRSGTSSGAGGSITRGAPVVVPELGKPRQSPLTYEDHGAPFTSHLP

1501 ARALERACYEESLKSRSGTSSGAGGSITRGAPVVVPELGKPRQSPLTYEDHGAPFTSHLP

1560 RGSPVTTREPTPRLQEGSLLSSKASQDRKLTSTPREIAKSPHSTVPEHHPHPISPYEHLL

1561 RGSPVTTREPTPRLQEGSLLSSKASQDRKLTSTPREIAKSPHSTVPEHHPHPISPYEHLL

1620 RGVTGVDLYRGHIPLAFDPTSIPRGIPLEAAAAAYYLPRHLAPSPTYPHLYPPYLIRGYP

1621 RGVTGVDLYRGHIPLAFDPTSIPRGIPLE-AAAAYYLPRHLAPSPTYPHLYPPYLIRGYP

1680 DTAALENRQTIINDYITSQQMHHNAASAMAQRADMLRGLSPRESSLALNYAAGPRGIIDL

1680 DTAALENRQTIINDYITSQQMHHNAASAMAQRADMLRGLSPRESSLALNYAAGPRGIIDL

1740 SQVPHLPVLVPPTPGTPATAIDRLAYLPTAPPPFSSRHSSSPLSPGGPTHLAKPTATSSS

1740 SQVPHLPVLVPPTPGTPATAIDRLAYLPTAPPPFSSRHSSSPLSPGGPTHLAKPTATSSS

1800 ERERERERERDKSILTSTTTVEHAPIWRPGTEQSSGAGGSSRPASHTHQHSPISPRTQDA

1800 ERERERERERDKSILTSTTTVEHAPIWRPGTEQSSGAGGSSRPASHTHQHSPISPRTQDA

1860 LQQRPSVLHNTSMKGVVTSVEPGTPTVLRSTSTSSPVRPAATFPPATHCPLGGTLEGVYP

1860 LQQRPSVLHNTSMKGVVTSVEPGTPTVLRSTSTSSPVRPAATFPPATHCPLGGTLEGVYP

1920 TLMEPVLLPKETSRVARPERPRVDAGHAFLTKPPAREPASSPSKSSEPRSLAPPSSSHTA

1920 TLMEPVLLPKETSRVARPERPRVDAGHAFLTKPPAREPASSPSKSSEPRSLAPPSSSHTA

1980 IARTPAKNLAPHHASPDPPAPTSASDLHREKTQSKPFSIQELELRSLGYHSGAGYSPDGV

1980 IARTPAKNLAPHHASPDPPAPTSASDLHREKTQSKPFSIQELELRSLGYHSGAGYSPDGV

2040 EPISPVSSPSLTHDKGLSKPLEELEKSHLEGELRHKQPGPMKLSAEAAHLPHLRPLPESQ

2040 EPISPVSSPSLTHDKGLSKPLEELEKSHLEGELRHKQPGPMKLSAEAAHLPHLRPLPESQ

2100 PSSSPLLQTAPGIKGHQRVVTLAQHISEVITQDYTRHHPQQLSGPLPAPLYSFPGASCPV

2100 PSSSPLLQTAPGIKGHQRVVTLAQHISEVITQDYTRHHPQQLSGPLPAPLYSFPGASCPV

2160 LDLRRPPSDLYLPPPDHGTPARGSPHSEGGKRSPEPSKTSVLGSSEDAIEPVSPPEGMTE

2160 LDLRRPPSDLYLPPPDHGTPARGSPHSEGGKRSPEPSKTSVLGSSEDAIEPVSPPEGMTE

2220 PGHARSTAYPLLYRDGEQGEPRMGSKSPGNTSQPPAFFSKLTESNSAMVKSKKQEINKKL

2220 PGHARSTAYPLLYRDGEQGEPRMGSKSPGNTSQPPAFFSKLTESNSAMVKSKKQEINKKL

2280 NTHNRNEPEYNIGQPGTEIFNMPAITGAG-----------------------------GG

2280 NTHNRNEPEYNIGQPGTEIFNMPAITGAGLMTCRSQAVQEHASTNMGLEAIIRKALMGGG

2311 GKAKVSGRPSSRKAKSPAPGLASGDRPPSVSSVHSEGDCNRRTPLTNRVWEDRPSSAGST

2340 GKAKVSGRPSSRKAKSPAPGLASGDRPPSVSSVHSEGDCNRRTPLTNRVWEDRPSSAGST

2371 PFPYNPLIMRLQAGVMASPPPPGLAAGSGPLAGPHHAWDEEPKPLLCSQYETLSDSE 2427

2400 PFPYNPLIMRLQAGVMASPPPPGLAAGSGPLAGPHHAWDEEPKPLLCSQYETLSDSE 2456

**Table S3.** Non-synonymous alterations commonly observed in both initial myelodysplastic syndromes (MDS) and relapse of acute myeloid leukaemia (AML)

| **Gene** | **Function** | **Single Nucleotide Variant or insertion/deletion** | **Amino acid change** |
| --- | --- | --- | --- |
| *MICAL3* | Missense | *c.4606G>A* | p.D1536N |
| *SH3BP4* | Missense | *c.2130G>C* | p.R710S |
| *MKI67* | Missense | *c.3152C>G* | p.T1051S |
| *GABRR3* | Missense | *c.262G>T* | p.D88Y |
| *ZNF674* | Missense | *c.796C>G* | p.Q266E |
| *CCT8L2* | Missense | *c.1031C>T* | p.P344L |
| *PUS1* | Missense | *c.1169G>C* | p.W390S |
| *PLXNB3* | Missense | *c.3350C>T* | p.A1117V |
| *LRIG2* | Missense | *c.1854G>A* | p.M618I |
| *FRMD4A* | Missense | *c.2640G>C* | p.K880N |
| *ARHGAP23* | Deletion | *c.3651_3653delGCC* | p.P1218del |
| *TCEA3* | Frameshift | *c.399_400insC* | p.K134fs |
| *KRAS* | Frameshift | *c.504delG* | p.L168fs |

**Table S4.** Non-synonymous alterations additionally observed only in relapse of acute myeloid leukaemia (AML)

| **Gene** | **Function** | **Single Nucleotide Variant** | **Amino acid change** |
| --- | --- | --- | --- |
| *E2F8* | Missense | *c.1133G>C* | p.C378S |
| *FECH* | Missense | *c.102G>C* | p.W34C |
| *B4GALNT1* | Missense | *c.827G>C* | p.S276T |
| *POLR3A* | Missense | *c.3713C>G* | p.T1238R |
| *ACP4* | Missense | *c.1072C>T* | p.P358S |
| *SCN2A* | Missense | *c.4114G>C* | p.G1372R |
| *C4orf50* | Missense | *c.3422G>C* | p.G1141A |
| *EPS8L2* | Missense | *c.74C>G* | p.A25G |
| *TRIL* | Missense | *c.1786G>C* | p.A596P |
| *MARCOL* | Missense | *c.631G>C* | p.G211R |
| *BAZ1A* | Missense | *c.1663C>G* | p.L555V |
| *GASK1A* | Missense | *c.1405C>G* | p.H469D |
| *DET1* | Missense | *c.730G>C* | p.V244L |
| *LAMA1* | Missense | *c.8521C>G* | p.L2841V |
| *PATL1* | Missense | *c.409G>C* | p.G137R |
| *VWA1* | Missense | *c.232G>C* | p.V78L |
| *TTI1* | Missense | *c.150G>T* | p.Q50H |
| *ZNF385D* | Missense | *c.1102A>G* | p.T368A |
| *BOD1L2* | Missense | *c.454G>C* | p.V152L |
| *PHGDH* | Missense | *c.130G>C* | p.E44Q |
| *SLC2A13* | Missense | *c.1296G>C* | p.Q432H |
| *LAT2* | Missense | *c.80G>A* | p.R27H |
| *JADE3* | Missense | *c.1982C>G* | p.S661C |
| *POU3F4* | Missense | *c.851C>T* | p.S284F |
| *CCDC88C* | Missense | *c.1965G>C* | p.E655D |
| *MUC16* | Missense | *c.34035G>C* | p.E11345D |
| *BMP6* | Missense | *c.353A>T* | p.Q118L |
| *CAPN8* | Missense | *c.335C>G* | p.A112G |
| *BCOR* | Nonsense | *c.3340G>T* | p.E1114* |

**Table S5.** List of common transcription regulatory area (TRA) alterations and neighbouring (within 20 kb) genes in order of the number of transcriptional regulators

| **Chr** | **Gene** | **transcriptional regulators** |
| --- | --- | --- |
| chr13  111617933  T>C | *LINC00431* | ATF2, ATF3, BATF, BCL11A, CEBPB, E2F4, EP300, FOS, FOSL1, FOSL2, FOXM1, GABPA, JUN, JUND, NFATC1, NFIC, RUNX3, POLR2A, IRF4, NR2F2, STAT5A, PML, MTA3, SMARCC1, MAX, TCF12, PAX5, BCL3, ATF1, MYC, MAFK, RFX5, STAT3, POU2F2, GATA2, RELA, MAFF, PBX3, SETDB1, SP1, RCOR1, CHD2, TRIM28, SMARCC2REST, ZBTB33, USF1, ARID3A, TCF7L2, YY1, MEF2C, E2F6, MEF2A, CCNT2, TBP, NFYA, NFYB |
| chr20  34359777  T>A | *PHF20* | CREB1, E2F1, E2F6, MAX, POLR2A, TAF1, CBX3, NR3C1, CEBPB, MYC, NRF1, MXI1, FOXP2, SETDB1, BHLHE40, EGR1, JUND, ATF1, TBP, RBBP5, E2F4, GATA3, SREBP1, HMGN3, SP4, PML, PAX5, ATF2, SIN3A, JUN, BRCA1, SP1, CHD2, FOS, MAZ, TBL1XR1, UBTF, IRF1, HDAC2, REST, RCOR1, RFX5, CHD1, GTF2F1, ELF1, STAT3, ELK1, GATA2 |
| chr2  231084719  G>C | *SP110* | ELF1, IRF1, POLR2A, PRDM1, SPI1, STAT1, STAT2, STAT3, RUNX3, PML, MTA3, MYC, KAP1, ZNF263, SIN3A, GABPA, MXI1, TAF1, STAT5A, ATF2, NFIC, TBP, MAX, FOXM1, POU2F2, SIN3AK20, MAZ, IRF4, SMC3, CHD2, FOS, ELK1, ELK4, FOXP2, WRNIP1 |
| chr1  184774936  C>G | *NIBAN1* | CTCF, RAD21, SMC3, FOXM1, ZNF143, YY1, RUNX3, POLR2A, TEAD4, MAZ, GATA3, JUND, POU2F2, PAX5, RCOR1, CBX3, FOS, ATF2, BCLAF1, MEF2A, RELA, CTCFL, FOXA1, BHLHE40, UBTF, RFX5, EP300, MAX, FOXP2, SIN3AK20, CREB1, E2F4 |
| chr10  102731069  C>T | *SEMA4G* | FOXA1, FOXA2, SP1, ZNF263, EP300, HNF4G, MYBL2, NFIC, POLR2A, MXI1, FOSL2, TAF1, YY1, SIN3AK20, REST, SRF, CEBPD, MAZ, JUND, TCF7L2, TCF12, TBP |
| chr5  108084826  G>T | *FER* | POLR2A, CTCF, MAZ, MAX, RBBP5, E2F4, MXI1, E2F1, SIN3A, TEAD4, TFAP2A, TFAP2C, EBF1, ZNF143, EP300, PAX5, RAD21, E2F6, SIN3AK20, JUN |
| chr1  36335340  C>G | *AGO4, AGO1* | BHLHE40, ELF1, MAX, POLR2A, EGR1, MYC, PHF8, E2F6, USF1, TBP, CEBPB, PML, GABPA, ATF3, SAP30, HDAC1, IRF1, ATF1, REST, SIN3AK20 |
| chr3  111837305  T>C | *C3orf52* | CTCF, POLR2A, RUNX3, ATF2, MTA3, EBF1, POU2F2, RAD21, SPI1, CHD1, SRF, YY1, MXI1, MAX, TBL1XR1, TCF12, WRNIP1 |
| chr13  67804865  G>C | *PCDH9* | E2F1, PHF8, POLR2A, MAZ, SIN3A, TAF1, RBBP5, SP4, MYC, KAP1, CTBP2, ELK4, GABPA, THAP1, TBP |
| chr9  132389168  C>G | *NTMT1* | POLR2A, ZNF263, HNF4A, HNF4G, CEBPB, NFATC1, YY1, SIN3AK20, TBP, CHD1, SP1, TAF1, ESRRA |
| chr10  22251103  A>T | *DNAJC1* | TEAD4, GATA2, MYC, JUND, CBX3, NR2F2, GATA1, STAT5A, MAZ, JUN, TBP |
| chr4  38426008  A>T | *LINC01258* | FOXA1, FOXA2, TEAD4, NFIC, EP300, ZBTB7A, HDAC2, JUND, RAD21, MYC, USF1 |
| chr6  137736958  C>A |  | CTCF, RAD21, SMC3, ZNF143, YY1, MTA3, RUNX3, CBX3, FOXM1, IRF4, CTBP2 |
| chr1  18908082  C>T |  | CTCF, RAD21, SMC3, ELF1, MAZ, ZNF143, GABPA, FOXP2, REST, POLR2A, TCF12 |
| chr9  4549409  del | *SLC1A1* | CTCF, RAD21, SMC3, YY1, RUNX3, PAX5, ZNF143, ELF1, SPI1 |
| chr15  67245780  T>G | *LINC02206* | USF1, BHLHE40, USF2, STAT3, MAX, BCL3, PBX3, NFYB |
| chr10  4284248  A>T | *LINC00702* | TCF7L2, POLR2A, TAF1, CEBPB, MAFK, RAD21, JUN, REST |
| chr4  123039786  del |  | TEAD4, EP300, GATA2, PML, STAT5A, TBL1XR1, CCNT2, POLR2A |
| chr8  21906359  A>G | *FGF17* | EZH2, POLR2A, E2F1, UBTF, RBBP5, CCNT2, E2F6 |
| chr7  1110306  C>T | *C7orf50* | POLR2A, STAT5A, MYC, BCL3, REST, CTCF, NR3C1 |
| chr3  35226539  C>T | *NR110817.1* | CEBPB, STAT3, EP300, FOS, RFX5, JUND, BRCA1 |
| chr14  22971834  A>C | *NR148361.1* | RUNX3, BATF, ATF2, EBF1, SPI1, PAX5, IRF4 |
| chr5  66308220  C>T | *MAST4* | FOS, STAT3, POLR2A, MYC, JUN, EP300 |
| chr2  224600694  T>C | *AP1S3* | MAFK, EP300, GATA2, FOS, CEBPB, TAF1 |
| chr11  105992407  C>T | *LINC02719* | EP300, FOXA1, TCF12, REST, GATA3, NR3C1 |
| chr5  137475288  C>T | *NME5* | SIN3A, MXI1, RUNX3, POLR2A, SIN3AK20 |
| chr12  106976201  G>T | *RFX4* | EZH2, BACH1, HDAC2, CHD1, RBBP5 |
| chr10  35930413  ins G | *FZD8* | CTCF, POLR2A, EZH2, CHD1, HMGN3 |
| chr3  176787890  A>T | *TBL1XR1* | FOS, CEBPB, JUND, EP300, POLR2A |
| chr3  142851496  A>G | *CHST2* | NFIC, ATF2, FOXM1, STAT5A, MTA3 |
| chr17  41690030  G>A |  | FOSL2, JUND, JUN, HNF4A, FOS |
| chr11  94296724  G>A | *FLT4, NR135093.1, PIWIL4, NR135096.1* | CTCF, JUND, FOS, JUN, ATF1 |

**Table S6.** List of additional transcription regulatory area (TRA) alterations and neighbouring (within 20 kb) genes in the order of the number of transcriptional regulators. Specific transcriptional regulators (RXRA, KDM5B, RCOR1, CHD2, PHF8, STAT1, GATA1, and HDAC1) are indicated in red.

| **Chr** | **Gene** | **transcriptional regulators** |
| --- | --- | --- |
| chr8  145582249  G>C | *FBXL6, SLC52A2* | EGR1, ELF1, GABPA, **PHF8,** POLR2A, TAF1, PML, E2F1, YY1, **HDAC1,** MAX, CREB1, MYC, MXI1, REST, **KDM5B**, SIN3A, PBX3, SP2, PAX5, ELK1, **CHD2,** POU2F2, FOXP2, RBBP5, TBP, MAZ, SAP30, E2F6, IRF1, SIN3AK20, RELA, NR3C1, SPI1, HDAC2, TBL1XR1, UBTF, SRF, JUND, TFAP2A, HMGN3, BACH1, CCNT2, E2F4, NFYA |
| chr1  1590495  T>A | *CDK11B* | ATF1, ELF1, GABPA, CREB1, POLR2A, ATF3, SRF, BRCA1, TBL1XR1, ELK1, MYC, SPI1, JUND, MAZ, TAF1, MAX, SIN3AK20, CBX3, **CHD2,** RUNX3, CEBPB, NR2F2, EP300, ELK4, SIX5, RBBP5, ZBTB33, ETS1, TBP, IRF1, THAP1, TAF7, RFX5, SIN3A, E2F4, JUN, **RXRA**, NRF1, MAFF, SETDB1, FOXP2, TCF7L2, BCLAF1, GRp20 |
| chr6  139309326  C>G | *REPS1* | ELF1, **PHF8,** POLR2A, YY1, TAF1, **CHD2,**  E2F1, TBP, KAP1, RBBP5, FOXM1, MAX, PML, REST, MAZ, MXI1, MYC, ZBTB33, HMGN3, E2F4, SIN3AK20, **KDM5B,** FOXP2, E2F6, TAF7, JUND, UBTF, **RCOR1,** BCLAF1, CREB1, **HDAC1,** SIN3A, BRCA1, GTF2F1, TCF12, CCNT2, ETS1, ELK1, **STAT1,** NR3C1 |
| chr6  14744335  C>G |  | CEBPB, FOS, FOSL1, FOSL2, JUN, JUNB, JUND, MAX, EP300, POLR2A, ATF3, RUNX3, MYC, NR3C1, SMARCC1, E2F6, BHLHE40, STAT3, RAD21, CHD2, NFIC, TCF7L2, MAZ, TCF12, E2F1, ATF2, SMC3, TAF1, TBP, GABPA, E2F4, ATF1, RCOR1, RFX5, SMARCC2, GATA2, EGR1, SMARCB1, GTF2F1, BCL3 |
| chr12  125402275  ins A | *MIR5188, UBC* | GABPA, POLR2A, RUNX3, TCF7L2, ELF1, **CHD2,** TAF1, MAX, BCL3, **PHF8,** IKZF1, TBP, NFIC, SIN3AK20, RELA, RBBP5, SRF, MEF2A, MAFK, **GATA1,** TEAD4, MAFF, MYBL2, CCNT2, EP300, FOXA1, SIN3A, FOSL2, IRF1, **HDAC1,** ZBTB7A, MEF2C, STAT1, JUND, JUN, SAP30, HSF1, GTF2B |
| chr2  96891008  T>G | *STARD7-AS1* | RELA, SPI1, GATA2, POLR2A, FOXM1, EP300, IKZF1, RUNX3, ZNF143, NFIC, NFATC1, IRF4, JUND, MEF2A, ATF2, IRF1, PML, **PHF8,** TEAD4, TBP, BCL11A, **RCOR1,** SP1, ELF1, **CHD2,** REST, YY1, MYC, STAT3, WRNIP1, TAF1, CCNT2, RXRA |
| chr20  47805239  C>G | *STAU1* | E2F1, POLR2A, SIX5, SPI1, ZNF143, **CHD2,** YY1, MAZ, MXI1, IRF1, MYC, E2F4, TBP, SP1, CBX3, USF2, CCNT2, SMARCC2, MYBL2, SIN3A, BCLAF1, SETDB1, MAX, E2F6, HMGN3, TBL1XR1, GTF2F1, **STAT1,** BRCA1, THAP1, STAT3 |
| chr2  69828348  G>C | *AAK1* | **GATA1,** GATA2, JUND, TCF7L2, EP300, JUNB, FOS, FOSL2, JUN, POLR2A, CEBPB, **RCOR1,** ARID3A, TBL1XR1, MAFK, TRIM28, CBX3, CCNT2, PML, NR2F2, STAT5A, MYC, GABPA, **STAT1,** MEF2A, RFX5, USF2, GTF3C2, SIRT6, **CHD2,** HMGN3 |
| chr7  6616884  del | *ZDHHC4* | GABPA, **PHF8,** POLR2A, TAF1, ELK1, MAX, **CHD2,** PML, MYC, MAZ, YY1, TBP, UBTF, CCNT2, ELK4, ATF2, TFAP2A, SMARCB1, NR2C2**, RCOR1, GATA1, RXRA,** NFYA, RFX5, **STAT1,** GTF2F1, HMGN3 |
| chr19  58341643  G>A | *ZNF587B* | CTCF, ABPA, POLR2A, TAF1, USF1, YY1  ZNF143, BHLHE40, USF2, SIX5, SIN3A, RAD21, TBP, ATF2, MAX, ELF1, TAF7, TEAD4, SP4, ATF3, SMC3 , MYC, SP1, CTCFL, **RXRA,** SIN3AK20 |
| chr9  136286982  C>G | *ADAMTS13* | RUNX3, CTCF, ZNF263, RELA, BHLHE40, POLR2A, ATF2, POU2F2, MXI1, EP300, TBP, WRNIP1, RAD21, SP1, PAX5, USF2, RFX5, **CHD2,** EGR1, MAX, SMC3, ELF1, TCF12, BATF |
| chr6  37016995  C>G |  | TAL1, YY1, RELA, JUND, STAT5A, MYC, BCL3, CHD1, EP300, TEAD4, POLR2A, KDM5B, PHF8, ETS1, GATA1, GATA2, ZNF143, HDAC2, TBL1XR1, HDAC1, TBP, SAP30, STAT1, SIN3AK20 |
| chr17  26971284  C>G | *KIAA0100* | CTCF, RUNX3, ELF1, GABPA, RAD21, TEAD4, ZNF143, POLR2A, SIN3AK20, MAX, JUND, SIN3A, SMC3, NR2F2, CHD1, MAZ, **RCOR1,** MYC, EP300, TCF12, YY1, TAF1, ELK4 |
| chr19 51298128  C>T | *ACP4* | CTCF, POLR2A, NFATC1, SIN3AK20, MTA3, YY1, BCL3, ELF1, ZNF263, JUND, **KDM5B, CHD2,** ATF2, ZBTB33, MAX, SIN3A, HMGN3, CREB1, EGR1, NR3C1, FOXP2, TAF1, CTBP2 |
| chr7 28995874  C>G | *TRIL* | CTCF, RAD21, **RXRA,** POLR2A, EZH2, NFIC, HNF4A, YY, HNF4G, ZNF143, MYC, GATA3, GABPA, HDAC2, MAX, ELF1, EP300, REST, **CHD2** |
| chr1 120254775  G>C | *PHGDH* | E2F1, POLR2A, TAF1, ZNF263, E2F4, **PHF8,** CHD1, **KDM5B,** TCF7L2, REST, SIN3AK20, MAZ, SIN3A, NR2C2, RBBP5, YY1, CTBP2 |
| chr14  95651879  C>G | *CLMN* | CEBPB, MAFK, MAFF, TEAD4, **RCOR1,** POLR2A, EP300, MAX, JUND, MYC, ATF3, STAT3, MAZ, BACH1, TCF3 |
| chr9  102981752  G>T | *INVS* | NFIC, RUNX3, SPI1, BATF, FOXM1, MEF2A, ATF2, MTA3, STAT5A, PML, IRF4, NFATC1, BCL11A, MEF2C, BCL3 |
| chr1  204168011  C>G | *GOLT1A* | CTCF, SMC3, RAD21, POLR2A, FOS, FOXA1, MYC, JUN, JUND, FOSL2, SIN3A, MAX, MXI1, TAF1 |
| chr2  47143478  ins G | *MCFD2* | E2F1, **PHF8,** POLR2A, YY1, ELK4, TAF1, PML, REST, TCF12, ETS1, TBP, MYBL2, PAX5 |
| chr15  31558215  C>T |  | GATA1, RUNX3, TCF3, POLR2A, TCF12, CTCF, BHLHE40, RXRA, MAX, MYC, ELF1, IRF4, ZEB1 |
| chr5  150618990  C>G | *CCDC69, GM2A* | CTCF, RAD21, SMC3, MAX, NF143, RUNX3, MYC, **RCOR1,** YY1, MAZ, TCF12, FOXP2 |
| chr10  102803098  G>A | *SFXN3, PDZD7,*  *KAZALD1* | POLR2A, ZNF263, E2F1, **PHF8,** PML, TAF1, MYBL2, SIN3A, REST, NFIC, TCF12, RBBP5 |
| chr10  48326329  C>A | *NR134500.1* | GATA2, FOS, FOSL2, JUND, NR3C1, MAX, POLR2A, FOXA1, CEBPB, EP300, TFAP2C, PRDM1 |
| chr6  142330903  C>G |  | TCF7L2, TEAD4, POLR2A, PRDM1, MAX, RCOR1, TAL1, GATA2, EP300, ATF1, ZBTB33, STAT3 |
| chr2  192419869  G>A |  | CTCF, SMC3, STAT3, ZNF143, STAT1, ELF1, RUNX3, YY1, CHD2, POU2F2, MYC |
| chr5  67702571  G>C |  | NR3C1, GATA1, MYC, NFATC1, NFIC, ZNF217, ATF2, TFAP2C, MXI1, TFAP2A |
| chr6  4378581  G>C |  | EP300, GATA2, CCNT2, JUND, MAZ, HMGN3, RCOR1, PML, MAX, MYC |
| chr20  29637650  C>T | *FRG1BP, MLLT10P1* | TCF7L2, NFIC, MAZ, IRF1, FOS, ELK1, MXI1, EZH2, IRF3 |
| chr10  25406856  G>T | *LINC01516* | JUND, EP300, MAFK, JUNB, **RCOR1,** ATF1, BHLHE40, CTCF, MAFF |
| chr22  44287608  C>T | *PNPLA5* | USF1, POLR2A, EZH2, EGR1, MXI1, MAX, CTCF, MYC, TBP |
| chr5  140810138  G>A | *PCDHGA12* | CTCF, RAD21, ZNF143, SMC3, MYC, CEBPB, ARID3A, MAX, YY1 |
| chr8  106545658  G>C | *ZFPM2* | CEBPB, TAL1, TEAD4, PML, STAT5A, EP300, NR2F2, GATA2, ARID3A |
| chr12  77266364  C>G | *CSRP2* | YY1, SIN3A, USF1, MXI1, MAX, POLR2A, POU2F2, SIN3AK20, MYC |
| chr11  69459465  C>G | *CCND1* | CTCF, POLR2A, MXI1, E2F1, NFIC, SP1, **RXRA,** NR3C1, YY1 |
| chr3  9773909  C>G | *BRPF1* | POLR2A, RBBP5, MYC, **HDAC1,** CHD1, SAP30, SMARCB1, SIN3AK20 |
| chr4  128446879  C>G |  | TAL1, TEAD4, EP300, STAT5A, GATA2, RCOR1, NR2F2, PML |
| chrX  9979678  ins AG | *WWC3* | RUNX3, ZZZ3, RELA, NFIC, EP300, MXI1, STAT1 |
| chr5  172197345  G>C | *DUSP1* | E2F1, EBF1, POLR2A, SIN3A, ZBTB33, SMARCB1, WRNIP1 |
| chr2  225503154  C>A |  | SPI1, NFATC1, KAP1, REST, SIN3A, MXI1, ELF1 |
| chr6  167854560  C>A |  | NANOG, YY1, TCF12, CHD1, SIN3A, JUND, MAX |
| chr21  44906144  C>G | *LINC00313* | CTCF, SMC3, MYC, RAD21, MAX, **RCOR1** |
| chrX  153606721  G>C | *EMD* | POLR2A, MYC, MAX, **HDAC1, KDM5B,** CHD1 |
| chr21  41692750  C>T | *DSCAM* | GATA3, POLR2A, ZNF217, TCF7L2, FOXA1, ESR1 |
| chr22  42310104  G>C | *SHISA8* | EZH2, CTCF, YY1, HDAC2, SUZ12, HDAC6 |
| chr11  82206178  G>A |  | CTCF, RAD21, FOXA1, MAFK, MAFF, FOXA2 |
| chr3  157812186  G>C | *SHOX2, RSRC1* | EZH2, POLR2A, RBBP5, TEAD4, CTBP2 |
| chr11  111262602  C>G | *POU2AF1* | RUNX3, BATF, NFIC, TCF3, TCF12 |
| chr9  115101183  C>G | *PTBP3* | STAT3, CEBPB, FOSL2, EP300, MYC |
| chr8  96224003  G>C | *LINC01298* | FOXA1, CEBPB, FOXA2, EP300, ARID3A |
| chr19  55998662  C>G | *NAT14* | POLR2A, FOS, TCF12, REST, JUN |
| chr20  56073587  C>G | *CTCFL* | SPI1, CTCF, RUNX3, MYC, EBF1 |
| chr11  134146453  C>G | *GLB1L3* | CTCF, EZH2, RBBP5, RAD21, CTBP2 |
| chr1  196330540  G>C | *KCNT2* | FOS, FOSL2, POLR2A, CEBPB, GATA2 |
| chr12  124882584  G>C | *NCOR2* | BHLHE40, **RCOR1,** ZNF143, FOXA1, CEBPB |
| chr4  16688081  C>G | *LDB2* | CEBPB, GATA2, POLR2A, FOS, MAFK |
| chr14  54446192  C>G |  | TCF7L2, EP300, ARID3A, FOXA1, SIN3A |
| chr6  7727541  A>T | *BMP6* | EZH2, POLR2A, CTCF, SUZ12, SIN3AK20 |
